# Evolution of eumalacostracan development—new insights into loss and reacquisition of larval stages revealed by heterochrony analysis

**DOI:** 10.1186/2041-9139-6-4

**Published:** 2015-03-11

**Authors:** Günther Joseph Jirikowski, Carsten Wolff, Stefan Richter

**Affiliations:** Institut für Biowissenschaften, Allgemeine und Spezielle Zoologie, Universität Rostock, Universitätsplatz 2, 18055 Rostock, Germany; Institut für Biologie, Vergleichende Zoologie, Humboldt-Universität zu Berlin, Philippstr. 13, Haus 2, 10115 Berlin, Germany

**Keywords:** Malacostraca, Muscle development, Larval development, Nauplius Larva, Egg Nauplius, Heterochrony, Phylogeny

## Abstract

**Background:**

Within Malacostraca (Crustacea), direct development and development through diverse forms of larvae are found. Recent investigations suggest that larva-related developmental features have undergone heterochronic evolution in Malacostraca. In the light of current phylogenetic hypotheses, the free-swimming *nauplius* larva was lost in the lineage leading to Malacostraca and evolved convergently in the malacostracan groups Dendrobranchiata and Euphausiacea. Here we reconstruct the evolutionary history of eumalacostracan (Malacostraca without Phyllocarida) development with regard to early appendage morphogenesis, muscle and central nervous system development, and determine the heterochronic transformations involved in changes of ontogenetic mode.

**Results:**

Timing of 33 developmental events from the different tissues was analyzed for six eumalacostracan species (material for Euphausiacea was not available) and one outgroup, using a modified version of *Parsimov*-*based genetic inference* (PGi). Our results confirm previous suggestions that the event sequence of *nauplius* larva development is partly retained in embryogenesis of those species which do not develop such a larva. The ontogenetic mode involving a *nauplius* larva was likely replaced by direct development in the malacostracan stem lineage. Secondary evolution of the *nauplius* larva of Dendrobranchiata from this ancestral condition, involved only a very small number of heterochronies, despite the drastic change of life history. In the lineage leading to Peracarida, timing patterns of *nauplius*-related development were lost. Throughout eumalacostracan evolution, events related to epidermal and neural tissue development were clearly less affected by heterochrony than events related to muscle development.

**Conclusions:**

Weak integration between mesodermal and ectodermal development may have allowed timing in muscle formation to be altered independently of ectodermal development. We conclude that heterochrony in muscle development played a crucial role in evolutionary loss and secondary evolution of a *nauplius* larva in Malacostraca.

**Electronic supplementary material:**

The online version of this article (doi:10.1186/2041-9139-6-4) contains supplementary material, which is available to authorized users.

## Background

Heterochrony—evolutionary change in timing of developmental events—is a central concept in understanding the diversity of animal form [1–3]. Investigations of sequence heterochrony have provided support for the important role of this mechanism in morphological evolution, e.g., in the case of ossification timing in amphibians [4–6], snakes [7], birds [8], mammals [9], internal organ development of amniotes [10], or limb development of tetrapods [11]. However, comparatively few investigations focus on heterochrony in invertebrate evolution [12–16]. Invertebrates, such as the crustaceans (the potential paraphyly of crustaceans has no impact to our study), display an enormous disparity in development, exemplified by the multitude of larval forms and life histories found throughout this group. Here we set out to explore the impact of sequence heterochrony on life history evolution of the crustacean group Malacostraca (‘higher crustaceans’).

**Figure 1:**
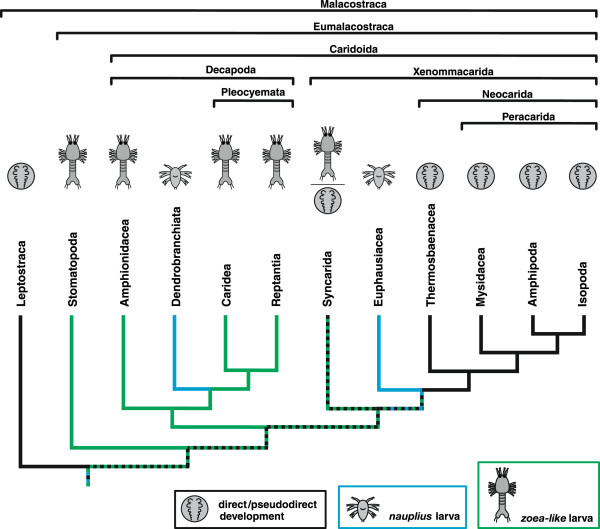
**Overview of malacostracan phylogeny.** Simplified representation of malacostracan phylogeny, following [28]. The major malacostracan monophyla suggested by these authors (Eumalacostraca, Caridoida, Decapoda, Pleocyemata, Xenommacarida, Peracarida) are marked with horizontal brackets. The taxa Anaspidacea and Bathynellacea are shown together as Syncarida. The peracaridan subtaxa Lophogastrida, Spelaeogriphacea, Mictacea, Tanaidacea, and Cumacea are excluded. Therefore, Amphipoda and Isopoda appear as sister groups. The developmental mode of the taxa is indicated by symbolic drawings at the bottom. The developmental mode is color coded to the branches and the most parsimonious character states of the ancestral lineages are shown. Outgroups are not depicted. Color coding: *Direct/pseudodirect development* (black); *nauplius larva as hatching stage* (*blue*); *zoea-like larva as hatching stage* (green).

Malacostraca represents a large and morphologically highly disparate taxon within crustaceans. Although malacostracans also have a rich fossil record, including larvae, we refer throughout our study to recent taxa only because musculature and nervous system is hardly known for fossil larvae or embryos. The plesiomorphic developmental mode generally accepted for crown-group Crustacea (or crown-group Tetraconata if crustaceans are paraphyletic in relation to Hexapoda) comprises hatching of a free-swimming, planktonic larva with conserved morphology, called *nauplius*[17–19]. The *nauplius* larva (i.e., *orthonauplius*) bears three pairs of appendages (first antenna, second antenna, and mandible), which are used for feeding and locomotion. In Malacostraca, such a larva is found only in two groups (Figure 1): Dendrobranchiata and Euphausiacea [20–23]. Moreover, despite controversies concerning the phylogenetic relationships within Malacostraca, Dendrobranchiata and Euphausiacea are always placed at nested positions within the tree [24–32]. In this light, the *nauplius* larva in Malacostraca has evolved secondarily from ancestors, which either showed direct development or hatched as a more advanced larval stage with a higher number of segments (Figure 1).

A variety of malacostracan taxa show yet another larval form that we call *zoea-like* larva (Figure 1). Contrary to the *nauplius* larva, the *zoea-like* larva is characterized by a complete or nearly complete number of body segments, a motile trunk including a paddle-shaped telson or tail fan. *Zoea-like* larvae are found in stomatopods (*pseudozoea*, *antizoea*) [20, 33–36], decapods (*mysis*, *zoea*) [37–43], bathynellaceans (*parazoea*) [44–46], and euphausiaceans (*calyptopis*) [47–50]. Stomatopod larvae of the *pseudozoea*-type have functional pleopods that are used for swimming. The *antizoea* larva of Lysiosquillidae (Stomatopoda) differs from the *pseudozoea* in that it swims using the throracic appendages and lacks pleopods. Following the phylogeny proposed by [51], the *pseudozoea* larva can be considered the ancestral condition for Stomatopoda. The *zoea-like* larvae of Decapoda, Euphausiacea, and Bathynellacea bear at least one pair of functional thoracopods, while the pleonal appendages are lacking.

Leptostraca, Anaspidacea (Syncarida), Thermosbaenacea, and Peracarida show different kinds of direct (or pseudodirect [12]) development and lack planktonic larvae (Figure 1) [52–58]. Direct development also evolved several times within the Decapoda, like the lineage leading to Astacidea which changed to a freshwater environment [28].

**Figure 2:**
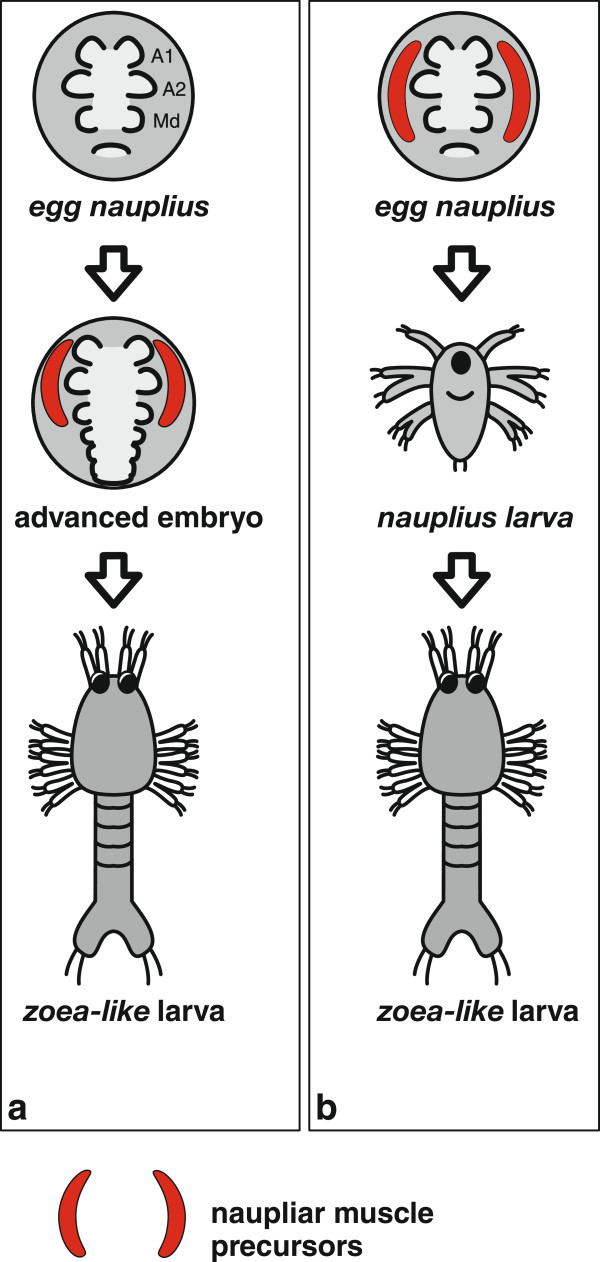
**Simple examples of malacostracan life histories.** Schematic representation of two Malacostracan life histories both involving a *zoea-like* larva. **(a)** Left: The larva is formed in embryogenesis, an *egg nauplius* stage is succeeded by an advanced embryonic stage with postnaupliar segments developed. **(b)** Right: The *zoea-like* larva is formed after preceding larval stages. An *egg nauplius* stage is traversed in both cases **(a and b)**. Naupliar musculature develops in the *egg nauplius* if a *nauplius* larva follows. Appendage buds are labeled only in the upper left *egg nauplius* drawing.

Leptostraca, Stomatopoda, Caridea (Decapoda), Reptantia (Decapoda), Anaspidacea, Bathynellacea, and Thermosbaenacea lack a *nauplius* larva but pass through a characteristic embryonic stage known as *egg nauplius*[59]. In the *egg nauplius*, the first antennal, second antennal, and mandibular buds (naupliar appendage buds) appear prior to the posterior (postnaupliar) appendage anlagen [44, 52, 53, 60–66]. Timing of naupliar and postnaupliar appendage bud formation is separated by a distinct gap. Scholtz [59] suggested that the *egg nauplius* is formed as part of a recapitulated developmental program originally involved in formation of a free-swimming *nauplius* larva. This *egg nauplius* concept has drawn our attention to the question how exactly transitions between larval and embryonic development are achieved in evolution. The presence of a larval developmental program in the malacostracan ground pattern can help to explain the secondary (and potentially independent) origin of the dendrobranchiate and euphausiacean *nauplius* larva [59]. Though this *egg nauplius* concept is of great value for understanding malacostracan evolution, it does not sufficiently consider developmental timing. For example, it treats the *egg nauplius* and the free *nauplius* larva as two alternative situations. Yet *nauplius* larvae are themselves preceded by embryonic stages which show three pairs of appendage buds (Figure 2). They differ from the *egg nauplius* stages of direct developers or species with *zoea-like* larvae, only by the lower amount of yolk and the more lateral position of the limb buds [67]. We prefer a more inclusive definition of the term ‘*egg nauplius*’ which applies also to all crustacean representatives with *nauplius* larvae. In our view, the *egg nauplius* represents a part of an ancestral developmental program which is shared between species with and without a free-swimming *nauplius* larva, before two different paths can be taken in development: (i) development of postnaupliar tissues, leading to a larger number of functional segments at hatching (Figure 2a) or (ii) differentiation and early functionality of the naupliar segments and hatching of a *nauplius* larva (Figure 2b).

The evolutionary scenario of recapitulated *nauplius* larva development in the *egg nauplius*[68] is largely based on gross external morphology of epidermal limb buds. Development of the nervous system or mesodermal tissue, such as musculature, has not played an important role, and only one publication discusses neurogenesis of a malacostracan species in an *egg nauplius* context [69]. Recently, we have found that anlagen of musculature develop in the naupliar segments only after the *egg nauplius* stage in several malacostracan representatives [67] (Figure 2a). The dissociated timing of mesodermal and ectodermal development suggests that retention of the ancestral larval developmental program does not occur in all tissues likewise and underwent heterochronic change in evolution. Here we will apply a developmental sequence approach to malacostracan development considering different tissue types (*epidermis*, *nervous tissue*, and *muscle tissue*) to gain a more fundamental understanding of the evolutionary changes to developmental timing which caused loss and reacquisition of larvae in malacostracan evolution.

Following Alberch [68], heterochrony affects only particular features of the organism, never the whole. In the case of the *egg nauplius*, we want to determine the evolutionary changes of developmental timing in different body regions and tissue types. Thus the relation between the modular organization of the developmental program and heterochronic evolution in Malacostraca is at the heart of our study. A conservative view on malacostracan developmental evolution would assume that all embryonic naupliar tissues develop in species without a *nauplius* larva by the same timing pattern as they would in *nauplius larva-*bearing species. In this case, in the malacostracan last common ancestor, a developmental path would be taken that accelerates tissue development in the postnaupliar segments relative to the naupliar segments after the *egg nauplius* stage. We will refer to the initial part of the developmental sequence, in which only developmental events of the naupliar segments occur (but none of the postnaupliar segments), regardless of the respective tissue type, as ‘*egg nauplius phase*.’ Transition of developmental events from the *egg nauplius phase* to later positions in development, would prevent formation of a viable *nauplius* larva. Such changes would have had to be reversed during secondary evolution of the *nauplius* larva in Dendrobranchiata and Euphausiacea.

**Figure 3:**
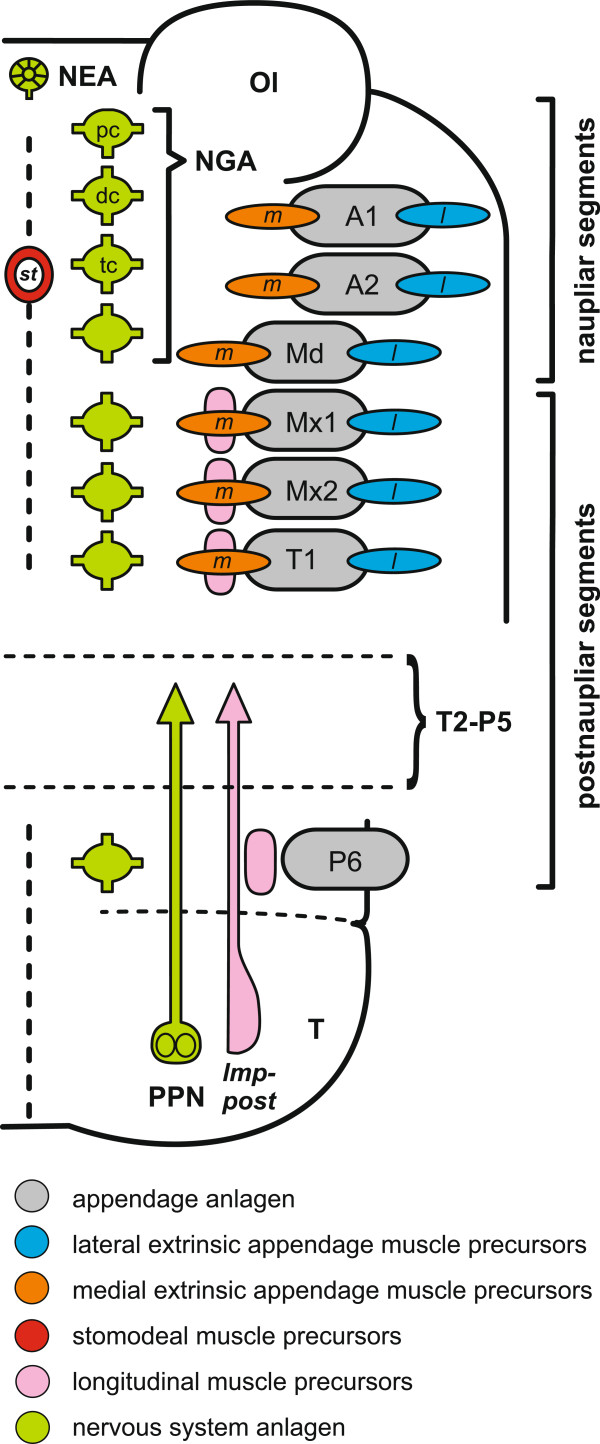
**Overview of investigated morphological features.** Schematic overview of malacostracan embryo or larva. The left hemisegments are shown in ventral view and hemisegments from the second thoracomere to the fifth pleomeres are excluded. A vertical dotted line at the left marks the longitudinal body axis. Anlagen of appendages, muscle precursor groups, and primordial elements of the nervous system are outlined and color-coded. The naupliar segments (bearing first antenna, second antenna, and mandible) and postnaupliar segments (all segments posterior to the mandible-bearing segment) are indicated by brackets on the right. Abbreviations: **Ol** optic lobe; **pc** protocerebral ganglion anlage; **dc** deutocerebral ganglion anlage; **tc** tritocerebral ganglion anlage; **NGA** naupliar ganglion anlagen; **NEA** nauplius eye anlage; **PPN** posterior pioneer neurons; **A1-P6** appendage anlagen of the first antennal to the sixth pleonal hemisegments; **T2-P5** excluded trunk segments; ***st*** stomodeal muscle precursor group, ***m*** medial extrinsic appendage muscle precursor groups; ***l*** lateral extrinsic appendage muscle precursor groups; ***lmp-post*** posterior longitudinal muscle precursor strand.

The morphological features to be investigated here in terms of developmental timing were chosen in a manner that allows comparison between tissue types, as well as between the germ layers ectoderm and mesoderm. Also, we rely on a large number of segmentally repeated features, namely appendage buds, ganglion anlagen, and muscle precursors to allow detection of timing differences also between segments. Such features are recorded for the head segments and the first trunk segment, the last trunk segment, and the telson (Figure 3). This allows us to record heterochronic changes in patterning of the naupliar and postnaupliar segments and to draw conclusions on their relation to loss or gain of a *nauplius* larva. Also features without obvious segment affiliation but with relevance to the evolution of *nauplius* larva development are included, such as the anlage of the nauplius eye, muscle anlagen of the stomodeum, and hatching from the egg envelope.

Other features are included which are potentially relevant for formation of a *zoea-like* larva and will serve to determine heterochronic changes that relate to this larval form: Formation of appendage buds, ganglion anlagen, and muscle precursors in the sixth pleonal segment, formation of a posterior longitudinal muscle precursor in the telson, and offset of segment formation. Offset of segment formation describes the point in development at which generation of body segments from the posterior growth zone terminates, and the full set of trunk segments is present as anlagen [67]. In Malacostraca, mesoderm and ectoderm of the trunk segments are formed by repeated asymmetric cell divisions of stem-like cells, the mesoteloblasts and ectoteloblasts, which are located in the growth zone in the posterior part of the embryo and can be recognized by the specific arrangement of stained nuclei. We use the mesodermal segment anlagen as reference for over all body segmentation here.

Timing of the first appearance (onset) of the specified features is recorded for six eumalacostracan representatives and one outgroup: *Gonodactylaceus falcatus* (FORSKÅL, 1775) (Stomatopoda), *Sicyonia ingentis* (BURKENROAD, 1938) (Dendrobranchiata), *Neocaridina heteropoda* (KEMP, 1918) (Caridea), *Procambarus fallax forma virginalis* (Astacidea), *Neomysis integer* (LEACH, 1814) (Mysidacea), *Parhyale hawaiensis* (DANA, 1853) (Amphipoda), and *Artemia franciscana* (KELLOGG, 1906) (Anostraca). *G. falcatus* hatches as a *zoea-like* larva (pseudozoea), *S. ingentis* hatches as *nauplius* larva, while the remaining species develop directly. A decapod representative that hatches as a *zoea-like* larva was not available. However, the late embryonic stages of *N. heteropoda* differ only little in morphology from other caridean *zoea* larvae. Thus, in terms of developmental timing, *N. heteropoda* can be considered a legitimate representative of *zoea*-bearing decapods. A representative of Euphausiacea could not be sampled for this study, because our methods demand fresh or appropriately fixed material and these animals are difficult to obtain. Thus we focus on the lineage leading to Dendrobranchiata to infer heterochrony related to evolution of a *nauplius* larva. *N. integer* differs from most other Peracarida in that it shows pseudodirect development. In this species, hatching occurs early in development, but the inert larva (*nauplioid*) remains in the brood pouch until juvenile morphology is established.

Based on the timing data, we apply a dynamic programming approach using the software *Parsimov-based genetic inference* (PGi) to trace evolution of the developmental sequences. The method used was first introduced by Harrisson & Larsson [70] and applied successfully in analyses of heterochrony since [4, 8, 71]. We have chosen the malacostracan phylogeny proposed by [28] and [72] as framework for the reconstruction of developmental evolution. It is in our view still the best supported one. We are aware that other suggested phylogenies [31] would give different results. The reconstruction of the ancestral developmental sequences of Eumalacostraca, Caridoida, Decapoda, Pleocyemata, and Peracarida, and inference of the heterochronic events that occurred along the different branches, will allow us to shed light on the evolutionary transformations of the segment and tissue-specific developmental processes that were involved in alteration of the developmental mode.

The following questions will be addressed (all taxon names refer to the respective crown-groups):

- *How did the last common ancestor (LCA) of Eumalacostraca develop and to what degree did it show larval developmental patterns?*
- *Which heterochronies were involved in evolution of the nauplius larva of Dendrobranchiata?*
- *Which changes of the developmental sequence caused the emergence of zoea-like larval forms?*
- *In which way were developmental sequences altered in the lineage leading to Peracarida?*

## Methods

### Specimen preparation, staining, and imaging procedure

Collection of embryo and larva material, fixation, fluorescent staining, and confocal microscopy followed by 3D image processing was performed in a previous investigation [67]. The respective methodology applied to *P. fallax forma virginalis* is described in [73]. Visualization of muscle tissue was performed on larval stages L4, L6, and L9 [74] of *A. franciscana*. The fixation protocol was previously described in [75]. Larvae of *A. franciscana* were incubated with Phalloidin-ALEXA 561 overnight to visualize muscle tissue by f-actin labeling. Imaris software Version 6.1 (Bitplane AG) was used to adjust image quality in projections of the volume data and to reconstruct and highlight single muscle precursors. Confocal image stacks of *S. ingentis* embryos and *nauplius* larvae labeled with BODIPY-FL-phallacidin were kindly provided by Phillip Hertzler for detection of nervous system development [76]. Embryos of 13h, 15h, 17h, and 20h after fertilization were analyzed, as well as *nauplius* stages 1 and 4. Immunohistochemical labeling of developing nervous tissue was performed on *G. falcatus*, *N. heteropoda*, *P. fallax forma virginalis*, and *N. integer* by application of an antibody against anti-acetylated α-tubulin (clone 6–11 B-1, Sigma T6793) which labels neurites, even at early developmental stages. For this the same preparation and staining, protocols as for muscle precursor labeling were used. Also histochemical staining with phalloidin-ALEXA488 (Molecular Probes, A12379) was applied to visualize developing ganglion anlagen of early embryonic stages. Graphics were drawn and image tables were assembled using CorelDRAW Graphics Suite X3 (Corel Corporation, Ottawa).

### Developmental sequence data

**Figure 4:**
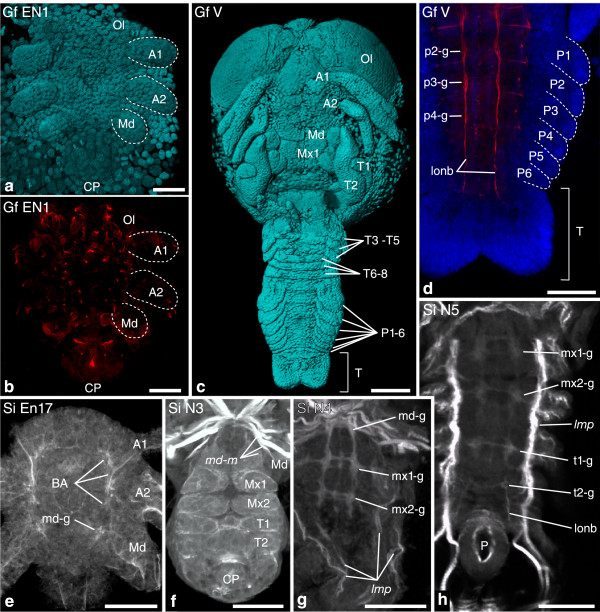
**Investigated morphological features of different semaphoronts of** ***G. falcatus*** **(Stomatopoda) and** ***S. ingentis*** **(Dendrobranchiata).** External morphology and developing nervous system of *G. falcatus* **(a–d)** and developing nervous system/muscular tissue of *S. ingentis* **(e–h)**. **(a)** Semaphoront **Gf EN1**, showing naupliar appendage buds. **(b)** Same semaphoront, showing acetylated alpha-tubulin signal. Ganglion anlagen are not yet visible. **(c)** Semaphoront **Gf V**, ventral view of complete embryo, showing anlagen of all appendages. **(d)** Same semaphoront, developing pleon and telson anlage. Ganglion anlagen are not developed posterior to the fourth pleomeres. Continuous longitudinal neurite bundles extend into the telson. **(e)** *S. ingentis* semaphoront **Si En 17**. Anlagen of naupliar ganglia are present in the embryo. **(f)** semaphoront **Si N3**. The larva shows four pairs of postnaupliar appendage buds. **(g)** Semaphoront **Si N4**. Ganglion anlagen are present in the first and second maxilla segment. **(g)** Semaphoront **Si N5**. Ganglion anlagen of the first and second thoracic segments are present. **(a, c)** Blend projection of nuclear signal (TOPRO-3) from confocal image stack; **(b)** maximum intensity projection of acetylated alpha-tubulin signal; **(d)** extended section of acetylated alpha-tubulin (red)- and TOPRO-3-signal (blue); **(e–h)** extended sections of phallacidin-BODIPY-labeled specimens, with permission of Phil Hertzler. Abbreviations: **BA** brain anlage, **Ol** optic lobes, **A1–P6** appendage anlagen of respective segments, **T** telson anlage, **pc** protocerebrum, **dc** deutocerebrum, tc tritocerebrum, **md-g**–**P6**-**g** ganglion anlagen of respective segments, **lonb** longitudinal neurite bundles, ***md-m*** medial extrinsic mandible muscle precursor, ***lmp*** longitudinal muscle precursor, **P** proctodeum, **CP** caudal papilla. Scale bars 50 μm in **(a**, **b**, **e–h)**; 200 μm in **(c)**, 100 μm in **(d)**.

**Figure 5:**
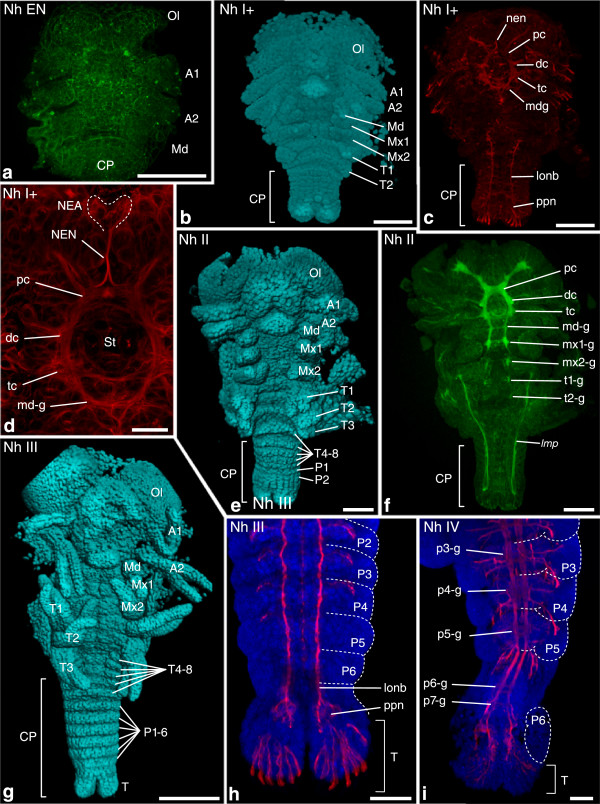
**Investigated morphological features of different semaphoronts of** ***N. heteropoda*** **(Pleocyemata).** External morphology and developing nervous system of *N. heteropoda*. **(a)** Semaphoront **Nh EN**, showing naupliar appendage buds, but no ganglion anlagen. **(b)** Semaphoront **Nh I+** showing postnaupliar appendage anlagen **Mx1–T1**. **(c)** Same semaphoront, showing naupliar ganglia and longitudinal neurite bundles. **(d)** Higher magnification image of the specimen shown in **(c)**. Anlagen of the protocerebrum, deutocerebrum, tritocerebrum, mandible ganglion and nauplius eye are visible. **(e)** Semaphoront **Nh II** showing postnaupliar appendage anlagen **T3** and additional posterior segment anlagen. **(f)** Same semaphoront showing postnaupliar ganglion anlagen from the first maxillary to the second thoracic segment. **(g)** Semaphoront **Nh III** showing the full set of segment anlagen. Pereiopod anlagen are present. **(h)** Same semaphoront, higher magnification of posterior pleon and telson anlage. No ganglion anlagen are observed in the pleonal segments, but continuous longitudinal neurite bundles extend anteriorly from the posterior pioneer neurons. **(i)** Semaphoront **Nh IV**, posterior pleon and telson anlage. Ganglion anlagen with extending lateral nerves are observed in all pleomeres, as well as a seventh pleonal ganglion anlage. **(a**, **c**, **d**, **f**, **h**, **i)** Maximum intensity projections of **(a**, **f)** phalloidin-signal (green), and **(c**, **d**, **h**, **i)** acetylated alpha-tubulin signal (red). In **(h)** and **(i)**, nuclei are shown in blue. Intersegmental furrows are demarcated by dotted lines. **(b)** Blend projection, **(e)** and **(i)** normal shading projection of nuclear signal (TOPRO-3) shown in cyan. Abbreviations: **Ol** optic lobes, **A1–P6** appendage anlagen of respective segments, **T** telson anlage, **pc** protocerebrum, **dc** deutocerebrum, **tc** tritocerebrum, **md-g**–**P6-g** ganglion anlagen of respective segments, **lonb** longitudinal neurite bundles, **NEA** nauplius eye anlage, **NEN** nauplius eye nerve, ***lmp*** longitudinal muscle precursor, **P** proctodeum, **CP** caudal papilla. Scale bars 100 μm in **(a–c**, **e**, **f**, **h**, **i)**; 25 μm in **(d)**; 500 μm in **(g)**.

**Figure 6:**
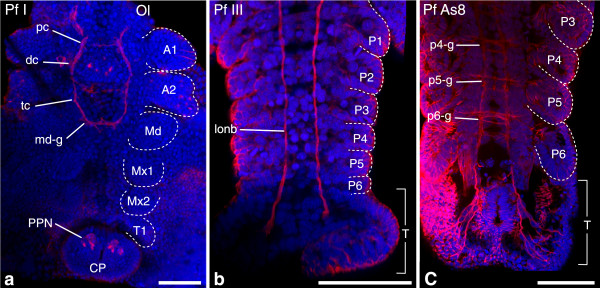
**Investigated morphological features of different semaphoronts of** ***P. fallax forma virginalis*** **(Pleocyemata).** Developing nervous system of *P. fallax forma virginalis*. **(a)** Semaphoront **Pf I**, ventral view of embryo. The naupliar ganglia are present, as well as posterior pioneer neurons [NGA], [PPN]. Appendage anlagen of the first antennal to the second thoracic segments are present. **(b)** Semaphoront **Pf III**, pleon and telson anlage. Ganglion anlagen are observed in none of the pleonal segments, but continuous longitudinal neurite bundles extend anteriorly from the posterior pioneer neurons. **(c)** Semaphoront **Pf As8**, posterior pleon and telson anlage. Developing ganglia are observed in all pleomeres, including the sixth pleomere [p6-g]. All panels show maximum intensity projections of acetylated alpha-tubulin signal (red) and nuclear signal (TOPRO-3), (blue), from confocal image stacks. In **(a–c)**, appendage anlagen are demarcated with dotted lines. Abbreviations: **Ol** optic lobes, **A1**- **P6** appendage anlagen of respective segments, **T** telson anlage, **pc** protocerebrum, **dc** deutocerebrum, **tc** tritocerebrum, **md-g**–**p6-g** ganglion anlagen of respective segments, **lonb** longitudinal neurite bundles, **CP** caudal papilla. Scale bars 100 μm in all panels.

**Figure 7:**
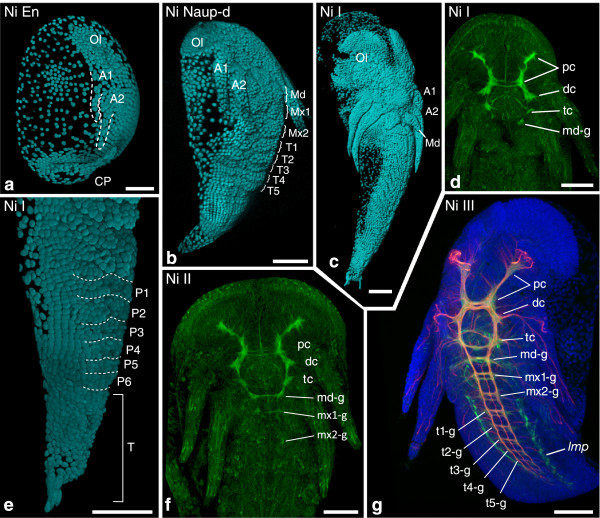
**Investigated morphological features of different semaphoronts of** ***N. integer*** **(Peracarida).** External morphology and developing nervous system of *N. integer*. **(a)** Semaphoront **Ni EN**, showing buds of the first and second antenna [A1/A2], but not the mandible. **(b)** Semaphoront **Ni Naup-d** showing mandible buds and postnaupliar appendage anlagen from the first maxilla to the fifth thoracic segment [Md], [Mx1], [Mx2], [T1]. **(c)** Semaphoront **Ni I**. The full set of segment anlagen is present, including the sixth pleomere. **(e)** Magnified lateral view of same semaphoront showing the posterior trunk and telson anlage. Appendage buds are visible down to the second pleomere. **(d)** Same semaphoront, showing phalloidin signal in naupliar ganglia [NGA]. **(f)** Semaphoront **Ni II**. Faint ganglion anlagen are visible also in the first and second maxillary segment [mx1-g], [mx2-g ]. **(g)** Semaphoront **Ni III**, with ganglion anlagen observable also from the first thoracomere [t1-g] down to the seventh thoracomere. The more anterior neuromeres show extensive formation of nerve fibers. The phalloidin signal also shows developing musculature. **(a, e)** blend projection, **(b, c)** normal shading projection of nuclear signal (TOPRO-3) from confocal image stacks (cyan). **(d**, **f)** Maximum intensity projections of phalloidin-signal (green). **(g)** Maximum intensity projections of phalloidin-signal (green), acetylated alpha-tubulin signal (red), and nuclear signal (TOPRO-3), (blue). Abbreviations: **Ol** optic lobes, **A1–P6** appendage anlagen of respective segments, **T** telson anlage, **pc** protocerebrum, **dc** deutocerebrum, **tc** tritocerebrum, **md-g**–**p6-g** ganglion anlagen of respective segments, **lonb** longitudinal neurite bundles, ***lmp*** longitudinal muscle precursor, **CP** caudal papilla. Scale bars 100 μm in all panels.

**Figure 8:**
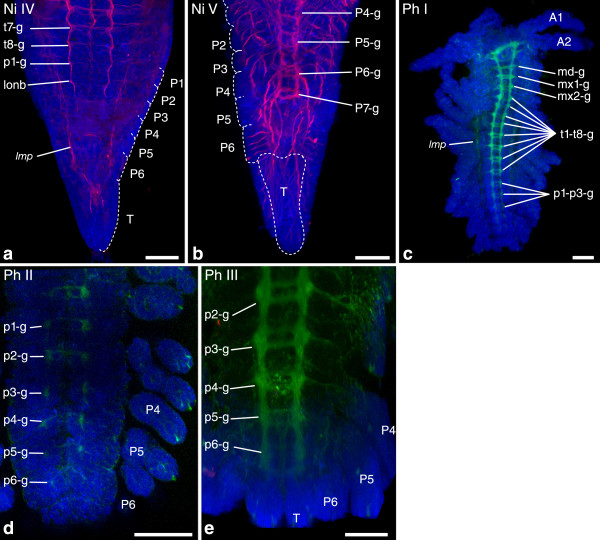
**Investigated morphological features of different semaphoronts of** ***N. integer*** **and** ***P. hawaiensis*** **(Peracarida).** Developing nervous system of *N. integer* and *P. hawaiensis*. **(a)** Semaphoront **Ni IV**. Ganglion anlagen are observable down to the first pleomeres. **(b)** Semaphoront **Ni V**. Ganglion anlagen and extending lateral nerves are observable in all pleomeres, including the sixth pleomeres [p6-g]. Also an additional seventh pleonal ganglion is seen. **(c)** Semaphoront **Ph I**. Overview of an entire embryo. Appendage buds are present in all segments. Ganglion anlagen are observable down to the third pleomere. **(d)** Semaphoront **Ph II**. Developing pleon. Faint ganglion anlagen are visible in the sixth pleomere [p6-g]. **(e)** Semaphoront **Ph III**. Ganglia of the pleonal segments are enlarged and show developing commissures. **(a, b)** *N. integer* **,** maximum intensity projections of acetylated alpha-tubulin signal (red) and nuclear signal (TOPRO-3), (blue). Dotted lines demarcate appendage anlagen. **(c)** *P. hawaiensis*, maximum intensity projection of phalloidin signal (green) and nuclear signal (TOPRO-3), (blue). **(d**, **e)** *P. hawaiensis*, extended confocal sections generated by the same staining procedure. Abbreviations: **A1–P6** appendage anlagen of respective segments, **T** telson anlage, **md-g**–**p6-g** ganglion anlagen of respective segments, ***lmp*** longitudinal muscle precursor. Scale bars 100 in **(a–c)**, 50 μm in **(d)** and **(e)**.

**Figure 9:**
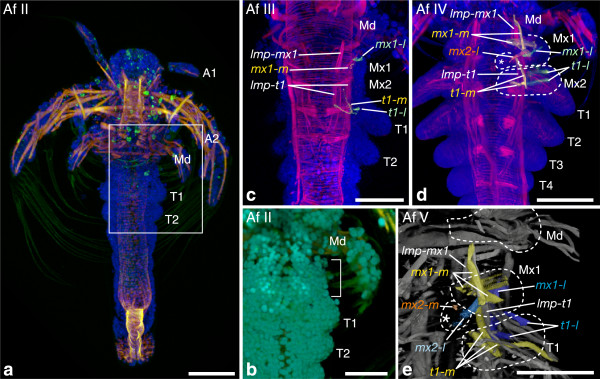
**Investigated morphological features of different semaphoronts of** ***A. franciscana*** **(Branchiopoda).** External morphology and developing musculature of *A. franciscana* larvae. **(a)** Overview of semaphoront **Af II**. Postnaupliar muscle precursors are not present, except for visceral musculature of the gut and proctodeum. **(b)** Higher magnification image of the region demarcated by a white rectangle in **(a)**. Appendage anlagen are seen in the first [T1] and second thoracic segment, but not in the first and second maxilla segment (demarcated with a bracket). **(c)** Semaphoront **Af III**. Appendage buds become visible in the first and second maxillary segment. Longitudinal muscle precursors extend from the first maxilla segment into the anterior thoracomeres [*lmp-mx1*], [*lmp-mx2*], [*lmp-t1*]. Medial and lateral extrinsic appendage muscle precursors appear in the first maxilla segment and the first thoracopod segment [*mx1-m*], [*mx1-l*], [*t1-m*], [*t1-l*]. **(d)** Semaphoront **Af IV**. A lateral extrinsic muscle precursor appears in the second maxillary segment [*mx2-l*]. **(e)** Semaphoront **Af V**. All muscle precursor groups show advanced differentiation and a medial extrinsic muscle precursor of the second maxilla has appeared [*mx2-m*]. **(a, c, d)** Maximum intensity projection of phalloidin-signal (red) and nuclear signal (TOPRO-3), (blue). In **(a)**, also autofluorescence signal of the cuticle is shown (green). **(b)** Blend projection of phalloidin-signal (orange) and nuclear signal (TOPRO-3), (light blue) and cuticle autofluorescence signal (green). **(e)** Blend projection of phalloidin-signal (light grey). In **(c, d, e)**, extrinsic appendage muscle precursors are reconstructed and highlighted. Medial muscle precursors are shown in yellow and orange, lateral extrinsic precursors are shown in blue and light blue. Abbreviations: **A1–T4** appendage anlagen of respective segments, ***lmp-mx1***, ***lmp-mx2***, ***lmp-t1*** longitudinal muscle precursors of the respective segments; ***md-m***, ***mx1-m***, ***mx2-m***, ***t1-m*** medial extrinsic appendage muscle precursors of the respective segments, ***md-l***, ***mx1-l***,***mx2-l***, ***t1-l*** medial extrinsic appendage muscle precursors of the respective segments. Scale bars are 100 μm in all panels.

The ontogeny of an individual organism can be viewed as an array of semaphoronts [77]. Hennig’s concept of the *semaphoront* has recently been revived to improve morphology-based phylogenetic inference on Pancrustacea/Tetraconata [78]. A semaphoront, in the sense of Hennig is ‘[…] the individual at a certain, theoretically infinitely small, period of its life’ [77, p6]. Sequences of developmental events always refer to series of semaphoronts. We will speak of semaphoronts instead of developmental stages throughout this paper, because (i) staging systems are not established for all of the species we investigate and (ii) staging systems rely on specific criteria that limit their resolution. For the present work however, we must allow timing to be recorded even within stages and based on new criteria, thus defining new operational stages that we refer to as semaphoronts. Developmental event sequences [79] were recorded for each species from the semaphoront series. Every event refers to the first appearance of a morphological feature (listed in Table 1). We restrict the present investigation to such ‘onset events’ to limit the size of the data set. The majority of events in our data set were coded from our previously published comparative study of malacostracan muscle development [67]. A substantial part of the event data was acquired from new observations presented in the results section (Figures 4, 5, 6, 7, 8, 9). In cases where data could not be provided by our own investigations, events were coded using the literature. An overview of these events and a list of the publications used are given in Table 2. A detailed overview of semaphoronts and events is given in Additional file 1. Early appendage morphogenesis of Astacidea and Amphipoda was coded from [80, 81], early neurogenic events for Stomatopoda, Astacidea, Amphipoda, and *Artemia sp*. were coded from [82–84], and early myogenic events for Dendrobranchiata and *Artemia sp*. were coded from [76, 84]. Features which were not recorded to be formed in development but are reported to be present in adults were coded as events at the end of the sequence. This is an effort to avoid missing data where possible, even if this results in artificial simultaneity between late events. This was the case for developmental sequences of all groups [85–91], except for *Artemia sp*. Following the position of the events in the sequence, ranks were assigned to every event (Table 3). An overview of the event series for all species, also showing stage specifications from the literature, rank values, and literature sources used for specific events, is provided in Additional file 1. Ranks ranged from 9 to 11, depending on the total number of semaphoronts described for each species.

**Table 1:** **Overview of events and event abbreviations**

Event group	Event	Information	Abbreviation
Epidermal appendage development	1	Anlage of the first and second antenna present.	[A1/A2]
	2	Anlage of the mandible present.	[Md]
	3	Anlage of the first maxilla present.	[Mx1]
	4	Anlage of second maxilla present.	[Mx2]
	5	Anlage of the first thoracopod present.	[T1]
	6	Anlage of the sixth pleopod present.	[P6]
Segmentation	7	All mesodermal segment anlagen present.	[FS]
Myogenesis	8	Stomodeal muscle precursor group present.	[*st*]
	9	Medial extrinsic appendage muscle precursor of first antenna present.	[*a1-m*]
	10	Lateral extrinsic appendage muscle precursor of second antenna present.	[*a1-l*]
	11	Medial extrinsic appendage muscle precursor of second antenna present.	[*a2-m*]
	12	Lateral extrinsic appendage muscle precursor of second antenna present.	[*a2-l*]
	13	Medial extrinsic appendage muscle precursor of mandible present.	[*md-m*]
	14	Lateral extrinsic appendage muscle precursor of mandible present.	[*md-l*]
	15	Medial extrinsic appendage muscle precursor of first maxilla present.	[*mx1-m*]
	16	Lateral extrinsic appendage muscle precursor of first maxilla present.	[*mx1-l*]
	17	Longitudinal muscle precursor in first maxilla segment present.	[*lmp-mx1*]
	18	Medial extrinsic appendage muscle precursor of second maxilla present.	[*mx2-m*]
	19	Lateral extrinsic appendage muscle precursor of second maxilla present.	[*mx2-l*]
	20	Longitudinal muscle precursor in second maxilla segment present.	[*lmp-mx2*]
	21	Lateral extrinsic appendage muscle precursor of first thoracopod present.	[*t1-m*]
	22	Medial extrinsic appendage muscle precursor of first thoracopod present.	[*t1-l*]
	23	Longitudinal muscle precursor in first thoracopod segment present.	[*lmp-t1*]
	24	Longitudinal muscle precursor in sixth pleopod segment present.	[*lmp-p6*]
	25	Posterior longitudinal muscle primordium present.	[*lmp-post*]
Neurogenesis	26	Anlagen of naupliar ganglia present.	[NGA]
	27	Anlagen of first maxilla ganglion present.	[mx1-g]
	28	Anlagen of second maxilla ganglion present.	[mx2-g]
	29	Anlagen of first thoracopod ganglion present.	[t1-g]
	30	Anlagen of sixth pleopod ganglion present.	[p6-g]
	31	Anlage of the nauplius eye present.	[NEA]
	32	Posterior pioneer neurons with anterior longitudinal neurite bundles present.	[PPN]
Hatching	33	Larva or Juvenile hatches from the egg membrane. End of embryogenesis.	[HAT]

**Table 2:** **Overview of event data taken from literature**

Taxon	Literature (same species)	Literature (related species)	Event abbreviation
*Artemia sp.*	[75]	[84]	[NGA], [mx1-g], [mx2-g], [t1-g], [NEA], [PPN]
		[84]	[A1/A2], [Md], [*st*], [*a1-m*], [*a1-l*], [*a2-m*], [*a2-l*], [*md-m*], [*md-l*], [NGA]
Stomatopoda	[67]		[Mx1], [Mx2], [T1], [P6], [FS], [*st*], [*a1-m*], [*a2-m*], [*a2-l*], [*md-m*], [*md-l*], [*mx1-m*], [*lmp-mx1*], [*mx2-l*], [*lmp-mx2*], [*lmp-t1*], [*lmp-p6*], [*lmp-post*], [HAT]
	[82]		[NGA], [mx1-g], [mx2-g], [t1-g], [NEA], [PPN],
		[85]	[*a1-l*], [*mx1-I*], [*mx2-m*], [*t1-m*] [*t1-l*],
		[60]	[p6-g]
Dendrobranchiata	-		-
	[76]		[A1/ A2], [Md], [*st*]*,* [*a1-m*]*,* [*a1-l*]*,* [*a2-m*]*,* [*a2-l*]*,* [*md-m*]*,* [*md-l*]*,* [*mx1-m*]*,* [*mx1-l*]*,* [*mx2-m*]*,* [*mx2-l*]*,* [*t1-l*]*,* [*t1-m*]*,* [HAT]
		[86]	[P6], [FS], [*lmp-p6*]
Caridea	[67]		[A1/A2], [Md], [Mx1], [Mx1], [Mx2], [T1], [P6], [FS], [*st*], [*a1-m*], [*a2-m*], [*a2-l*], [*md-m*], [*md-l*], [*mx1-m*], [*mx1-l*], [*lmp-mx1*], [*mx2-m*], [*mx2-l*], [*lmp-mx2*], [*t1-m*], [*t1-l*], [*lmp-t1*], [*lmp-p6*], [*lmp-post*], [HAT]
		[87]	[*a1-l*]
Astacidea	[67]		[FS], [*st*], [*a2-l*], [*md-m*], [*md-l*], [*mx1-m*], [*mx1-l*], [*lmp-mx1*], [*mx2-m*], [*mx2-l*], [*lmp-mx2*], [*t1-m*], [*t1-l*], [*lmp-t1*], [*lmp-p6*], [*lmp-post*], [HAT]
	[69]		[A1], [A2], [Md], [Mx1], [Mx2], [T1], [P6]
	[80]		[NGA], [Mx1-g], [Mx2-g], [t1-g]
		[88]	[*a1-m*], [*a1-l*], [*a2-m*]
Mysidacea	[67]		[A1/ A2], [Md], [Mx1], [Mx1], [Mx2], [T1], [FS], [*st*], [*a2-m*], [*a2-l*], [*md-m*], [*md-l*], [*mx1-m*], [*mx1-l*], [*lmp-mx1*], [*mx2-m*], [*mx2-l*], [*lmp-mx2*], [*t1-m*], [*t1-l*], [*lmp-t1*], [*lmp-p6*], [*lmp-post*], [HAT]
		[89]	[*a1-m*], [*a1-l*]
		[90]	[NEA]
Amphipoda	[67]		[*st*], [*a2-m*], [*a2-l*], [*md-m*], [*md-l*], [*mx1-m*], [*mx1-l*], [*lmp-mx1*], [*mx2-m*], [*mx2-l*], [*lmp-mx2*], [*t1-m*], [*t1-l*], [*lmp-t1*], [*lmp-p6*], [*lmp-post*], [HAT], [FS]
	[81]		[A1/ A2], [Md], [Mx1], [Mx2], [T1], [P6]
		[83]	[NGA], [mx1-g], [mx2-g], [t1-g] [PPN]
		[91]	[*a1-m*], [ *a1-l*]
		[90]	[NEA]

The table gives an overview of literature sources which were used to determine developmental timing of specific events that could not be determined from our investigations, for each taxon used in the analysis. Event abbreviations are used as in Table 1.

**Table 3:** **Rank table of developmental sequences**

Event number	Abbreviation	***A.f.***	***G.f.***	***S.i.***	***N.h.***	***P.f.***	***N.i.***	***P.h.***
1	[A1/ A2]	2	1	1	1	1	1	1
2	[Md]	2	1	1	1	1	3	1
3	[Mx1]	7	3	7	3	2	3	2
4	[Mx2]	7	4	7	3	2	3	2
5	[T1]	6	4	7	3	2	3	2
6	[P6]	n.a.	7	11	7	6	5	5
7	[FS]	n.a.	5	10	4	4	3	4
8	[*st* ]	1	4	7	2	2	5	6
9	[*a1-m*]	2	5	3	4	9	9	11
10	[*a1-l*]	2	10	3	9	9	9	11
11	[*a2-m*]	2	7	4	2	9	7	8
12	[*a2-l*]	2	4	2	4	2	7	8
13	[*md-m*]	2	5	4	2	2	5	8
14	[*md-l*]	2	4	3	2	2	6	9
15	[*mx1-m*]	7	8	9	5	7	6	8
16	[*mx1-l*]	7	10	9	5	2	7	9
17	[*lmp-mx1*]	7	7	8	5	3	6	-
18	[*mx2-m*]	9	10	9	5	7	6	8
19	[*mx2-l*]	8	5	9	5	5	7	8
20	[*lmp-mx2*]	-	5	8	4	3	4	-
21	[*t1-m*]	7	10	9	5	7	7	8
22	[*t1-l*]	7	10	9	5	7	7	8
23	[*lmp-t1*]	7	4	8	4	3	4	6
24	[*lmp-p6*]	n.a.	7	10	5	5	6	9
25	[*lmp-post*]	-	4	8	4	3	-	-
26	[NGA]	2	2	3	2	2	4	3
27	[Mx1-g]	7	5	8	4	3	5	3
28	[Mx2-g]	7	5	8	4	3	5	3
29	[T1-g]	7	6	9	4	4	6	3
30	[P6-g]	n.a.	9	10	6	6	8	7
31	[NEA]	3	2	5	3	-	-	-
32	[PPN]	5	2	5	2	2	-	-
33	[HAT]	4	10	6	8	8	2	10

The table shows rank values assigned to the events coded for heterochrony analysis. Events are ordered by number and sorted into groups as in Table 1 and Table 2. Abbreviations of events are given in brackets. Rank values specify the position of the respective event in the event sequence for each of the investigated species. Rank values range from 1 to 11. Minus signs are given where the respective feature is not present in the species of interest. Non applicable events are marked ‘n.a.’. Abbreviations: *A.f.* (*A. franciscana*, Branchiopoda), *G.f*. (*G. falcatus*, Stomatopoda), *S.i.* (*S. ingentis*, Dendrobranchiata), *N.h*. (*N. heteropoda*, Caridea), *P.f*. (*P. fallax forma virginalis*, Astacidea), *N.i.* (*N. integer*, Mysidacea), *P.h*. (*P. hawaiensis*, Amphipoda). Event abbreviations are used as in Table 1.

The table gives a list of all events coded for comparative analysis of malacostracan development. The events specify the first appearance of a specific morphological structure (e.g.) appendage primordium or property (e.g.) hatched larva. Muscle precursor terminology was adapted from [58] for myogenic events. Abbreviations of muscle precursor groups are given in italics. The morphological features are sorted by the following categories: *Epidermal appendage development*, *segmentation*, *myogenesis*, *neurogenesis*, and *Hatching*. Events are numbered from 1–33 and this order is maintained for the analysis. Descriptions are given for every event, as well as abbreviations. Abbreviations of developmental events are given in brackets and used consistently throughout the paper.

### Heterochrony analysis

Ranks were coded as character states for the respective events in a matrix of 7 × 33 cells and exported as a NEXUS file (Additional file 2) together with the phylogeny from [28], using the open source software package Mesquite 2.75 [92]. The tree was simplified by excluding all taxa that are not represented in our sampling. We use *A. franciscana* as an outgroup. Since a free-swimming *nauplius* larva is found throughout the ‘entomostracan’ crustaceans, it must also have been present in the linage leading to the crown-group Malacostraca. We added the developmental sequence of *A. franciscana* twice to the matrix to allow optimization of this condition in the analysis. Analysis of heterochrony was performed using a modified version of PGi [69], kindly provided by Luke Harrisson. The method uses a dynamic programming approach which treats the event sequence as a single complex character. Therefore, it avoids the assumption of event independence which is inherent to event pair-based methodology of heterochrony analysis [93]. PGi uses a simplified genetic algorithm-based heuristic on the event sequence, and Parsimov event pairing [94] is used as edit cost function. The program runs in the *ape* package [95] and was carried out using the open source statistics environment ‘R’ (version 3.0.1) [96]. We performed three runs with the following parameters for the PGi simplified genetic algorithm: 100 cycles of selection per node, 200 sequences per cycle of selection, and a maximum of 100 ancestral developmental sequences to be retained at each node. For each run, the most parsimonious solutions of equal cost are collected by the algorithm and used to calculate a pseudoconsensus tree. Heterochronies that occur in the equally parsimonious solutions are included in the pseudoconsensus tree if they fulfill the 50% majority rule criterion and the percentage of each heterochrony is given as bootstrap support [69]. The pseudoconsensus method was set to ‘semi exhaustive’ and the limit of evaluated solutions of equal score was set to 3,000. The pseudoconsensus trees of the three independent runs were combined to a superconsensus tree (Additional file 3). The ancestral sequences are constructed by PGi from mean ranks that are calculated from the multiple equally parsimonious solutions of the pseudoconsensus trees [69]. Because of the high variation in the data set, the reconstructed ancestral sequences can show slight differences in event position that are not given as heterochronies by the analysis. Calculations were carried out on a Dell Optiplex790—computer with an i3-2100 CPU@3.1GHz and 8GB RAM, running 64Bit Microsoft Windows 7. Graphic representations of the superconsensus tree and transformation of developmental sequences were edited using CorelDRAW Graphic Suite X3.

For all events, heterochrony rates were calculated. The heterochrony rate of an event in our case represents the number of heterochronic changes recovered by PGi for that event in the superconsensus tree, multiplied by its mean bootstrap value. Tissue-specific heterochrony rates were calculated which represent the mean heterochrony rate per event for all events specific to epidermis, neural tissue, or muscle tissue development. These values were also used to determine mean heterochrony rates for the germ layers ectoderm and mesoderm. Likewise heterochrony rates were compared between segments and tabulated. For this, events with problematic segment affiliation (FS, *st*, NEA, and HAT) were excluded. Two events represent combinations of several segment-specific events ([A1/A2], [NGA]). In these cases, the heterochrony rate of the combined event is used for each of the single segments, because simultaneity is observed in each of the investigated species and can thus be assumed also for the ancestral sequences. Where multiple events are affiliated with the same segment (myogenic events), the mean heterochrony rate of these events is used.

## Results

### Recorded events

Event sequences were assembled for *A. franciscana*, *G. falcatus*, *S. ingentis, N. heteropoda*, *P. fallax forma virginalis*, *N. integer*, and *P. hawaiensis* by combination of our previous descriptions, literature data, and new observations presented here. Table 2 gives an overview of the literature sources used and of the events coded from them. The new observations are depicted in Figures 4, 5, 6, 7, 8, 9. Furthermore, specifications of literature sources and reference to the corresponding figures depicting the new observations in the present work are shown for each event in Additional file 1. Information on development of other species is used in several cases to complete the semaphoront sequence. In Additional file 1, these species are also shown for the respective events.

*Epidermal morphogenetic events* (1–6): The events recorded for epidermal morphogenesis represent the appearance of distinct appendage buds of the first antenna, second antenna, mandible, first maxilla, second maxilla, first thoracopod, and sixth pleopod. An event is scored when the appendage anlage is recognizable as protuberance in the epidermal layer. Formation of first and second antenna is scored as a single (event 1) [A1/A2] because they are always observed to occur simultaneously.

*Myogenic events* (8–25): First appearance of the muscle precursors described in [67] is recorded here as myogenic events. For the present investigation, we reduced the total number of muscle precursors by combining some precursors to groups (Table 1): The *stomodeal* muscle precursors are combined to one group (event 8). The same applies to the medial extrinsic appendage muscles of the first and second antenna, mandible, first and second maxilla, and first thoracopod, respectively (events 9, 11, 13, 15, 18, 21), as well as the lateral extrinsic appendage muscles of the same body segments (events 10, 12, 14, 16, 19, 22). First appearance of a group is registered as a developmental event, if any of the muscle precursors of one group is seen. Furthermore, the first appearance of longitudinal muscle precursors in the first maxilla, the second maxilla, and the first thoracopod segment (events 17, 20, 23) are recorded, but longitudinal muscle precursors of the mandible segment are excluded, as they occur only transiently in *G. falcatus* and *P. fallax forma virginalis*. Longitudinal muscle precursors of the sixth pleomeres (event 24) are recorded. They are recognizable as metameric, non-continuous muscle precursors in the sixth pleopod segment. The posterior longitudinal muscle primordium (event 25) represents a portion of the longitudinal muscle strand that extends posteriorly into the growth zone and telson anlage. This event is lacking in Peracarida and is coded as absent for *N. integer* and *P. hawaiensis*.

*Neurogenetic events* (26–32): Data that are lacking in the published material for *G. falcatus*, *P. fallax forma virginalis*, as well as data on neurogenesis of *N. heteropoda*, *N. integer*, and *P. hawaiensis* were obtained by the methodology described above. We specified eight events for development of the nervous system (Table 1), six of which relate to the first appearance of ganglion anlagen. Ganglion anlagen are defined here as metameric cellular arrangements in the neuroectoderm, with a developing central neuropile [97]. Developing nerve fibers of commissures, connectives and lateral nerves can be present. Ganglion anlagen which are preceded by longitudinal neurite bundles originating from the posterior pioneer neurons are recognizable as spindle-shaped regions formed by these longitudinal fibers as shown by [82]. We specified the first appearance of ganglion anlagen in the naupliar, the first maxillary, second maxillary, first thoracic segment (events 26–29), and in the sixth pleomere (event 30). Formation of the naupliar ganglia (protocerebrum, deutocerebrum, tritocerebrum, mandibular ganglion) are scored as a single event [NGA] because they are always observed to occur simultaneously. Developing ganglia are observed also posterior to the sixth pleomeres, e.g., a seventh pleonal ganglion in *N. heteropoda* or *N. integer*. For our purpose, we will record only the emergence of the sixth pleonal neuromere. Furthermore, we record the presence of the anlage of the nauplius eye, a feature commonly present in crustacean *nauplius* larvae (event 31), and of posterior pioneer neurons (event 32).

*Overall development and segmentation* (7, 33): We specified two features which are relevant for over all segment formation and differentiation: Offset of segment formation (event 7), recognizable by the presence of mesodermal segment anlagen of all thoracic and pleonal segments, and hatching from the egg membrane, which is at the same time the end of embryogenesis, is coded (event 33).

### Description of developmental sequences

For convenience, we use specific font style for event and semaphoront abbreviations throughout this paper. Semaphoront abbreviations are given in bold letters and contain a two-letter code for the species name, followed by a roman number specifying the position of this semaphoront in the sequence, or by an abbreviation of an established stage name. The semaphoront abbreviations are adapted from [67]. For hatching individuals, the abbreviation ‘**HAT**’ is used instead of the roman number. Abbreviations that refer to specific developmental stages that were coded from the literature are given in italics in brackets. The abbreviations for ontogenetic events are given in square brackets throughout this paper. In the following section, the coded semaphoronts of the investigated species are described for *G. falcatus*, *S. ingentis*, *N. heteropoda, P. fallax forma virginalis*, *N. integer*, *P. hawaiensis*, and *A. franciscana*, together with the respective developmental events.

*Gonodactylaceus falcatus:* The first semaphoront **Gf EN1** (Figure 4a) shows small buds of the first antenna, second antenna, and mandible [A1], [A2], [Md]. Acetylated α-tubulin-immunohistochemical labeling shows a signal scattered throughout the embryo, but it cannot be specifically assigned to ganglion anlagen (Figure 4b). The terminal cellular processes of the posterior pioneer neurons are detectable, but since the longitudinal neurite bundles are not yet seen, we did not assign [PPN] to this stage. **Gf EN2** possesses an enlarged caudal papilla, as well as anlagen of the protocerebral, deutocerebral, tritocerebral, and mandibular ganglia, anlagen of the nauplius eye, and the posterior pioneer neurons [NGA], [NEA], [PPN] (Additional file 1). **Gf I** shows the appendage bud of the first maxilla [Mx1]. In **Gf II**, the second maxillary and first thoracic appendage buds appear [Mx2], [T1], as well as the muscle precursors [*st*], [*a2-l*], [*md-l*], [*lmp-t1*] and [*lmp-post*]. In **Gf III**, the first postnaupliar ganglion anlagen are present, namely in the first and second maxilla segment [mx1-g], [mx2-g]. Furthermore, muscle precursors [*a1-m*], [*md-m*], [*mx2-l*], [*lmp-mx2*] arise and offset of segmentation [FS] is recorded. **Gf P2** represents an intermediate stage between **Gf III** and **Gf IV** and is characterized by the appearance of a ganglion anlage in the first thoracopod segment [t1-g]. In **Gf IV**, the appendage bud of the sixth pleopod arises [P6], as well as the muscle precursors [*a2-m*], [*lmp-mx1*] and [*lmp-p6*]. External morphology of **Gf V** is shown in Figure 4c. Developing nervous tissue of the pleon is shown in Figure 4d. [p6-g] is assigned to a novel semaphoront **Gf VI**. Formation of a lateral extrinsic appendage muscle precursor [*a1-l*], extrinsic appendage muscle precursors of the first maxilla and first thoracopod [*mx1-I*], [*t1-l*], medial extrinsic muscle precursors of the second maxilla, and the first thoracopod [*mx2-m*], [*t1-m*] could not be observed during development. Since these features are reported for the adult (Table 2, Additional file 1), we assign them to a final semaphoront **Gf HAT**, together with the hatching event [HAT].

*Sicyonia ingentis*: The initial semaphoront ***Si EN13*** represents the embryo at *13hpf* (13 h post fertilization) which shows distinct buds of the first antenna, the second antenna, and the mandible [A1], [A2], [Md] (Additional file 1). In the next semaphoront ***Si En15*** (*15hpf*), the first muscle precursors become detectable in the second antenna segment [*a2-l*]. In ***Si EN17*** (*17hpf*), anlagen of the protocerebral, deutocerebral, tritocerebral, and mandible ganglia appear [NGA] (Figure 4e). Additional muscle precursors arise in the first antennal and the mandibular segment [*a1-m*, *a1-l*, *md-l*]. The remaining naupliar appendage muscle primordia [*a2-m*], [*md-m*] appear in semaphoront ***Si En20*** (*20hpf*). ***Si EN23*** (*23hpf*) is characterized by distinct nerve fiber bundles forming the circumesophageal ring, the presence of the nauplius eye primordium [NEA] and the posterior pioneer neurons [PPN]. **Si N1** (*nauplius stage 1*) hatches from the egg membrane [HAT]. In **Si N3** (*nauplius stage 3*), appendage buds of the fist maxilla, second maxilla, first thoracopod, and second thoracopod appear [Mx1], [Mx2], [T1] (Figure 4f). ***Si N4*** (*nauplius stage 4*) shows anlagen of postnaupliar ganglia (Figure 4g) in the first and second maxilla segment [mx1-g], [mx2-g], already showing commissures. The same semaphoront also shows longitudinal muscle precursors, extending from within the first maxilla segment into the telson anlage. [*lmp-mx1*], [*lmp-mx2*], [*lmp-t1*], and [*lmp-post*] are therefore assigned to ***Si N4. Si N5*** (*nauplius stage 5*) shows ganglion anlagen in the first and second thoracopod segment [t1-g], (Figure 4h), as well as musculature associated with the postnaupliar appendages which we identify as extrinsic appendage muscle precursors [*mx1-m*], [*mx1-l*], [*mx2-m*], [*mx2-l*], [*t1-m*], [*t1-m*]. In the second *protozoea* stage, ***Lv Z2*** of *L. vannamei* segmentation of the trunk is complete, meaning all segmental mesoderm anlagen must have been formed [FS]. In a related species, *Penaeus monodon*, ganglion anlagen are present in the entire trunk [p6-g] at this stage. [P6], [FS], [*lmp-p6*], and [p6-g] are assigned to this semaphoront. In the second mysis stage, ***Lv M2*** appendage anlagen are present in the sixth pleomere [P6].

*Neocaridina heteropoda*: The *egg nauplius* stage, semaphoront **Nh EN**, shows first antennal, second antennal, and mandibular buds [A1], [A2], [Md], but no clear sign of differentiating nervous tissue (Figure 5a). Anti-acetylated α-tubulin staining data for **Nh I** could not be obtained. An additional semaphoront **Nh I+** is described here which shows intermediate limb morphology, between **Nh I** and **Nh II**: [Mx1], [Mx2], [T1], (Figure 5b). **Nh I+** also shows a differentiated circumesophageal nerve ring, an anlage of the nauplius eye, and posterior pioneer neurons with elongate longitudinal neurite bundles (Figure 5c,d). Since the anlagen of the naupliar ganglia must be formed between **Nh EN** and **Nh I+,** we assign the event [NGA] to **Nh I**. Because of the advanced morphology of the posterior pioneer neurons and longitudinal neurite bundles, [PPN] is also assigned to semaphoront **Nh I** while [NEA] is assigned to **Nh I**+. **Nh II** shows distinct buds of the third thoracopod (Figure 5e). The full set of mesodermal segment anlagen is present [FS]. Ganglion anlagen are present in the first and second maxilla segment and in the first and second thoracopod segments [mx1-g], [mx2-g], [t1-g]. The first maxilla segment shows developing commissures (Figure 5f). Semaphoront **Nh III** shows intersegmental furrows throughout the entire trunk (Figure 5g). Also more postnaupliar ganglia show differentiated commissures. However, the complete set of ganglion anlagen of the trunk is not yet present, though continuous longitudinal neurite bundles bilaterally connect the posterior pioneer neurons to the anterior ganglia (Figure 5h). Semaphoront **Nh IV** shows fully developed ganglia, in the most posterior trunk segments, including a sixth and an additional seventh pleomere ganglion anlage (Figure 5i). Therefore, we assign the feature [p6-g] to an intermediate semaphoront between **Nh III** and **Nh IV**: **Nh III** +. **Nh IV** shows appendage buds in the sixth pleon segment [P6]. The next semaphoront **Nh HAT** hatches from the egg envelope. Throughout embryogenesis of *N. heteropoda*, even in freshly hatched individuals, lateral extrinsic muscle precursors of the first antenna [*a1-l*] were not observed. They are assigned to a final semaphoront **Nh VI**.

*Procambarus fallax forma virginalis*: **Pf EN** represents the *egg nauplius* stage, with first antennal, second antennal, and mandibular buds [A1], [A2], [Md] (Additional file 1). In semaphoront **Pf I**, the embryo shows appendage buds of the first and second maxillae, the first thoracopod as well as anlagen of the naupliar ganglia [Mx1], [Mx2], [T1], [NGA]. Also posterior pioneer neurons [PPN] (Figure 6a) and a set of cephalic muscle precursors [*st*], [*a2-l*], [*md-m*], [*md-l*], [*mx1-l*], appear. **Pf II** shows distinct appendage buds down to the fifth thoracomere. Here early anlagen of ganglia in the first and second maxilla segments are described [mx1-g], [mx2-g] (Additional file 1), as well as the longitudinal muscle precursors [*lmp-mx1*], [*lmp-mx2*], [*lmp-t1*], [*lmp-post*]. These features are followed by the appearance of appendage buds of ganglion anlagen in the first thoracomere [t1-g] and offset of segment formation [FS] at **Pf AS6** (*AS06 stage 6*, *V06 45%*). In **Pf III**, ganglion anlagen of the most posterior trunk segment are still lacking, but continuous longitudinal neurite bundles are already present (Figure 6b). Two muscle precursors are formed: [*mx2-l*] and [*lmp-p6*]. Presence of the ganglion anlagen in pleomere six [p6-g] is assigned to semaphoront **Pf AS8**, (Figure 6c) which also shows presence of appendage buds in pleomere six [P6]. In **Pf VI**, further muscle precursors are formed: [*mx1-m*], [*mx2-l*], [*t1-m*], [*t1-l*]. Hatching of the juvenile [HAT] is assigned to semaphoront **Pf HAT**. [*a1-m*], [*a1-l*], and [*a2-m*] were assigned to a final semaphoront **Pf V**. The anlage of a nauplius eye [NEA] was never observed and is also not reported for adult crayfish. It is coded as absent.

*Neomysis integer*: The first semaphoront **Ni En** represents an embryonic semaphoront with slender anlagen of the first and second antenna [A1], [A2], but no mandible bud (Figure 7a). It is followed by the hatching event [HAT], assigned to semaphoront **Ni HAT** which again is followed by the first nauplioid stage, **Ni Naup**-**d** (Figure 7b). This semaphoront shows appendage buds of the mandibles, first and second maxillae and first thoracopods [Md], [Mx1], [Mx2], [T1], as well as the complete set of mesodermal segment anlagen [FS]. The following semaphoront **Ni I** shows external segmentation of the complete trunk (Figure 7c,e), as well as ganglion anlagen in the naupliar segments [NGA], (Figure 7d), as well as longitudinal muscle precursors in the second maxilla segment and the first thoracic segment: [*lmp-mx2*] and [*lmp-t1*]. **Ni II** shows stomodeal muscle precursors [*st*] and precursors of the medial mandible muscles [*md-m*]. Presence of the differentiated circumesophageal nerve ring formed by the naupliar ganglia, as well as anlagen of the first postnaupliar neuromeres down to the second maxilla segment [mx1-g], [mx2-g], can be seen (Figure 7f). These neuromeres, as well as more posterior ones show differentiated commissures and connectives in the next semaphoront **Ni III** (Figure 7g). [t1-g] is assigned to this semaphoront, as well as the remaining longitudinal trunk muscle precursors [*lmp-mx1*], [*lmp-p6*], and [*md-l*], [*mx1-m*], [*mx2-m*]. Semaphoront **Ni IV** shows further muscle precursors of the cephalic and first thoracic segments [*a2-m*], [*a2-l*], [*mx1-l*], [*mx2-l*], [*t1-m*], [*t1-l*], but no ganglion anlagen in pleomere six (Figure 8a). [p6-g] is assigned to an intermediate semaphoront **Ni IV**+, as semaphoront **Ni V** possesses a sixth (and seventh) neuromere showing connectives and lateral nerves with advanced differentiation (Figure 8b). Formation of the first antennal muscle precursors [*a1-m*], [*a1-l*] was not recorded during embryonic and larval development. These features are assigned to a final semaphoront **Ni VI**. Posterior pioneer neurons, a nauplius eye anlage, and posterior longitudinal muscle primordium [PPN], [NEA], [*lmp-post*] were neither observed nor are they reported for adult semaphoronts. They are coded as absent.

*Parhyale hawaiensis*: In the first semaphoront **Ph E1**, the first antennal, second antennal, and mandibular buds [A1], [A2], [Md] appear. Semaphoront **Ph E2** shows appendage buds in the first maxillary, second maxillary, and first thoracopod segments [Mx1], [Mx2], [T1]. Appearance of the naupliar, first maxillary, second maxillary, and first thoracic ganglion anlage [NGA], [mx1-g], [mx2-g], [t1-g], is reported for a corresponding semaphoront (*S3* early) of the amphipod *Orchestia cavimana*[83] and is assigned to semaphoront **Ph E3**. These ganglion anlagen appear rapidly in anterior posterior progression, with a slight gap between naupliar and postnaupliar segments. However, since these events all occur within a single stage and since single events do not coincide with different events of our series, we assign them to one semaphoront and treat them as simultaneous in the sequence. In semaphoront **Ph E4**, the germ band shows the full number of adult mesodermal segment anlagen [FS]. Appendage buds in the sixth pleon segment are seen in semaphoront **Ph E5** [P6]. In **Ph I**, the first muscle precursors appear: [*st*], [*lmp-t1*]. Ganglion anlagen of the ventral nerve cord are visible down to the third pleon segment (Figure 8c). The sixth pleomere shows early ganglion anlagen in semaphoront **Ph II** [p6-g], (Figure 8d). Ganglia of the sixth pleomere show differentiated commissures in semaphoront **Ph III** (Figure 8e). This semaphoront also shows several muscle precursors: [*a2-l*], [*md-m*], [*md-l*], [*mx1-m*], [*mx2-m*], [*mx2-l*], [*t1-m*], [*t1-l*]. Further muscle precursors arise in semaphoront **Ph IV**: [*md-l*]*,* [*mx1-l*], [*lmp-p6*]. Semaphoront **Ph V** is excluded from our semaphoront series, as it does not introduce novel features. The next semaphoront **Ph HAT** is characterized by hatching from the egg envelope. The final semaphoront **Ph VI** is assigned features that are not observed throughout development but are reported for the adult. [*a1-m*], [*a1-l*]. [*lmp-mx1*], [*lmp-mx2*], [*lmp-post*], [PPN], and [NEA] are neither observed during embryogenesis nor described for adult Amphipoda. They are coded as absent.

*Artemia franciscana*: **Af EN1** (*Na2*) is characterized by first appearance of stomodeal muscle precursors [*st*] (Additional file 1). **Af EN2** (*Na3*) shows first appearance of naupliar appendage buds, naupliar ganglia, as well as the naupliar appendage muscle primordia [A1], [A2], [Md], [NGA], [*a1-m*], [*a1-l*], [*a2-m*], [*a2-*]*l*, [*md-m*], [*md-l*]. We specify a third embryonic semaphoront **Af EN3**, to which we assign the appearance of the nauplius eye [NEA], according to published data (Additional file 1). **Af HAT** is characterized by hatching from the egg membrane. The first larval semaphoront **Af I** possesses posterior pioneer neurons [PPN]. **Af II** is characterized by appendage buds in the first thoracic segment [T1] (Figure 9a). Appendage buds in the first and second maxilla segments are not yet found at this stage (Figure 9b). Formation of ganglion anlagen in the first maxilla, second maxilla, and first thoracopod segments, [mx1-g], [mx2-g], [t1-g], are assigned to a novel semaphoront **Af III**. Here also first and second maxillary appendage buds appear [Mx1], [Mx2], as well as medial and lateral extrinsic appendage muscle precursors and longitudinal muscle precursors of the trunk [*mx1-m*], [*mx1-l*], [*t1-m*], [*t1-l*], [*lmp-mx1*], [*lmp-t1*] (Figure 9c). In the next semaphoront **Af IV**, a lateral extrinsic muscle precursor in the second maxilla segment [*mx2-l*] has emerged (Figure 9d). A small medial extrinsic muscle precursor of the second maxilla [*mx2-m*] is seen in semaphoront **Af V** (Figure 9e). A longitudinal muscle precursor of the second maxilla segment is not observed at any time in development. We code [*lmp-mx2*] as absent for *A. franciscana*. Furthermore, all features that relate to the sixth pleon segment [P6], [*lmp-p6*], [p6-g], also [*lmp-post*] and offset of segment formation [FS] are coded as absent, because these features are applicable only to Malacostraca.

### Heterochrony analysis and ancestral developmental sequences

**Figure 10:**
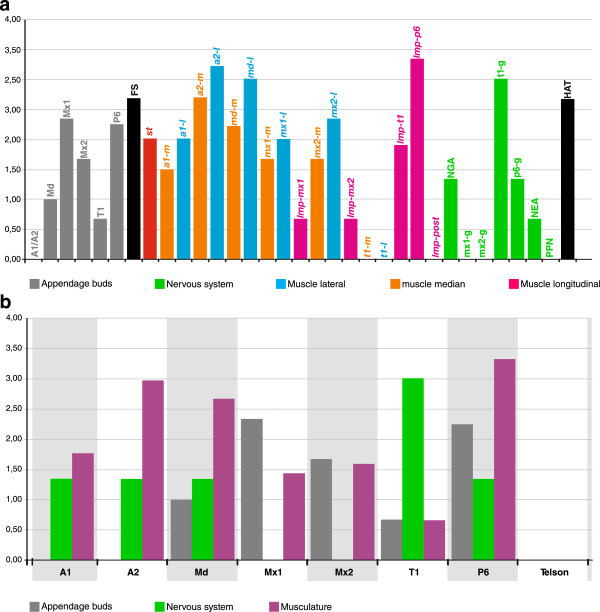
**Comparison of heterochrony rates.** Box graph showing heterochrony rates of all events in the superconsensus tree. The heterochrony rate represents the total number of heterochronic shifts (early and late) for a specific event, multiplied with its mean bootstrap value. The color code for the different classes of events is given at the bottom. **(a)** Heterochrony rate for all events in the data set. **(b)** Heterochrony rate of events with clear segment affiliation, sorted from anterior to posterior (A1, A2, Md, Mx1, Mx2, T1, P6) and compared for the three different tissue types (appendage buds, ganglion anlagen, and muscle precursors). The heterochrony rates shown here for muscle precursors represent mean values of all myogenic events recorded for the respective segment. Events that are affiliated with the telson (PPN, *lmp-post*) are also included. Abbreviations for events in **(a)** are used as listed in Table 1. In **(b)**, the body segments are labeled with the abbreviations of the corresponding appendage: **A1** first antenna, **A2** second antenna, **Md** mandible, **Mx1** first maxilla, **Mx2** second maxilla, **T1** first thoracopod, and **P6** sixth pleopod.

The single runs of PGi provided pseudoconsensus trees with lengths of 101, 100, 98, and a mean tree length of 99.67. Heterochrony in the eumalacostracan tree is extensive, with an average of 7.12 event changes per branch. This represents approximately 22% of the listed events. The superconsensus tree including ancestral and terminal sequences, as well as listed heterochronic events and bootstrap support is shown in Additional file 3. For all events, heterochrony rates were calculated (Figure 10). Formation of the first and second antenna buds [A1/A2], the extrinsic appendage muscle precursors of the first thoracopod [*t1-m*], [*t1-l*], the posterior pioneer muscle strand [*lmp-post*], the ganglion anlagen of the first and second maxilla segment [mx1-g], [mx2-g], and the posterior pioneer neurons [PPN] do not show any heterochrony (Figure 10a). In the case of extrinsic appendage muscle precursor formation, the lateral muscle precursors show a higher heterochrony rate than the medial muscle precursors. Comparison of segment specific heterochrony rates (Figure 10b) shows that myogenic events in the naupliar segments (A1, A2, Md) have been altered in evolution significantly more often than appendage bud and ganglion formation. The first and second maxilla segments show no heterochrony in neurogenesis but slightly higher rates of heterochrony in appendage bud formation than in myogenesis. Between tissue types the mean heterochrony rates differ strongly, as reflected by mean rates of 1.32, 0.91, and 1.72 changes per event for epidermis (appendage buds), nervous tissue and musculature, respectively (Table 4). This results in considerably differing heterochrony rates between germ layers, namely 1.12 in the ectoderm and 1.72 in the mesoderm.

**Table 4:** **Comparison of heterochrony rates between germ layers and tissue types**

Germ layer	Tissue type	Events	Mean heterochrony rate	Mean heterochrony rate of germ layer
Ectoderm	Epidermis	6	1.32	1.12
	Neural tissue	7	0.91	
Mesoderm	Muscle tissue	18	1.72	1.72

The table shows event numbers and heterochrony rates for the three tissue types from which the events were coded: Epidermis, muscle tissue, and nervous tissue, as well as the germ layers the tissues originate from (ectoderm, mesoderm). The heterochrony rate of an event is the event-specific number of heterochronic changes shown by PGi for the entire superconsensus tree, multiplied by the mean bootstrap value for the event. Tissue-specific heterochrony rates were calculated by forming the mean heterochrony rate per event for all events of epidermis neural tissue or muscle development. Mean heterochrony rates of the germ layers were calculated from these values.

In the following, the ancestral developmental sequences and heterochronies represented in the PGi superconsensus tree are presented.

#### The branchiopod/malacostracan last common ancestor

**Figure 11:**
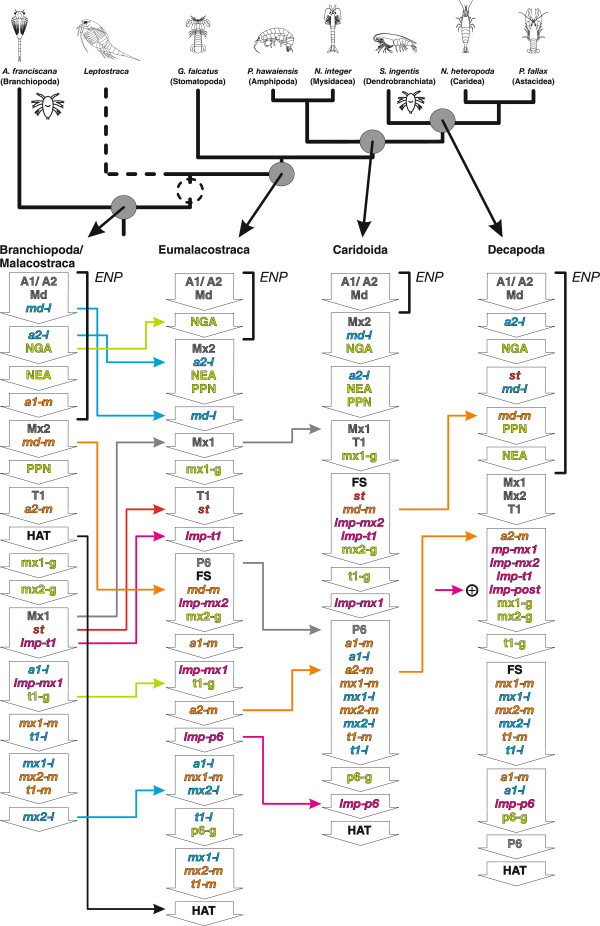
**Heterochronic changes and ancestral developmental sequences for major malacostracan nodes.** Simplified phylogram of Malacostraca with ancestral developmental sequences from the PGi superconsensus tree. Sequences are shown as columns of downward pointing arrows. Each arrow represents a different semaphoront containing a single event or a group of simultaneous events (abbreviations listed in Table 1). The position of Leptostraca is indicated by a dotted line. Ancestral ontogenetic sequences of the branchiopod/malacostracan, the eumalacostracan, the caridoid, and the decapod LCA are shown. Events are color-coded corresponding to Figures 3 and 10a. Heterochronic changes are indicated by horizontal arrows that use the same color code as the respective events. The *egg nauplius phase* is indicated by brackets. A symbolic *nauplius* drawing marks the terminal taxa, which develop *nauplius* larvae. Arrows with a ‘plus sign’ mark events that are interpreted as new evolutionary acquisitions on the respective branch. Arrows with a ‘minus sign’ indicate evolutionary loss of a feature. Abbreviations: ***ENP*** *egg nauplius phase*. Event abbreviations are listed in Table 1.

The analysis revealed an ancestral sequence for the branchiopod/malacostracan clade in which the naupliar appendage buds [A1/A2], [Md], the naupliar ganglion anlagen [NGA], and the nauplius eye anlage [NEA] are formed in the *egg nauplius phase*. However, this phase lacks a large part of the naupliar myogenic events: [*md-m*], [*a2-m*], [*a1-l*], and [*st*] (Figure 11). The hatching event [HAT] occurs only after the formation of the postnaupliar appendage buds [Mx2] and [T1]. The postnaupliar appendage buds and ganglion anlagen appear with significant delay to their counterparts in the naupliar segments. Furthermore, the postnaupliar appendage buds do not appear in anteroposterior progression in the reconstructed branchiopod/malacostracan LCA sequence. Buds of the second maxilla [Mx2] are formed first, followed by the first thoracopod bud [T1] and the first maxilla bud [Mx1].

#### Eumalacostraca

PGi analysis shows several heterochronic changes in the eumalacostracan stem lineage. [*a2-l*] and [*md-l*] are shifted out of the naupliar phase (Figure 11). The *egg nauplius phase* of the eumalacostracan LCA comprises only naupliar appendage bud formation [A1/A2, Md] and formation of naupliar ganglion anlagen [NGA]. Furthermore, [NGA] is shifted late within the *egg nauplius phase*. Formation of the mandibular muscle precursors [*md-m*] is shifted late while formation of the first maxilla bud [Mx1], the first thoracic ganglion anlage [t1-g], the stomodeal muscle precursors [*st*], and the longitudinal muscle precursor of the first thoracic segment [*lmp-t1*] are shifted early. The hatching event [HAT] is shifted to the end of the sequence. Even though formation of the nauplius eye anlage [NEA] and posterior pioneer neurons [PPN] are no longer part of the *egg nauplius phase* (which is defined by the absence of any postnaupliar events), they still occur near the beginning of the sequence. This position is not altered on the branches leading to Caridoida and Decapoda.

#### Caridoida

In the caridoid stem lineage formation of the first maxilla bud [Mx1], the sixth pleopod bud [p6] and the longitudinal muscle precursor [*lmp-p6*] are shifted late while formation of the second antenna muscle precursor [*a2-m*] is shifted early. The *egg nauplius phase* of the reconstructed caridoid LCA consists only of naupliar appendage bud formation [A1/A2, Md]. The first maxilla bud [Mx1] is still formed after the second maxilla bud [Mx2] while postnaupliar ganglia maintain their anteroposterior progression. Formation of appendage buds [P6], ganglion anlagen [p6-g], and longitudinal muscle precursors [*lmp-p6*] are now concentrated near the end of the sequence.

#### Decapoda

In the lineage leading to the decapod LCA, two naupliar myogenic events have shifted early [*md-m*] and [*a2-m*] (Figure 11). The *egg nauplius phase* comprises naupliar appendage bud formation [A1/A2, Md], the naupliar neural events [NGA, NEA], and the majority of naupliar myogenic events [*st*], [*a2-l*], [*md-l*], [*md-m*]. It is followed by the simultaneous formation of the first maxillary, second maxillary, and first thoracic appendage buds. The *egg nauplius phase* appears extensive in the sequence, due to the simultaneous occurrence of [Mx1, Mx2, T1]. However, the late position of [Mx2] was not revealed as heterochrony by the analysis, but results from the mean rank calculation for the superconsensus tree. The posterior longitudinal muscle precursor [*lmp-post*] is given as novelty for Decapoda by the analysis. Nevertheless, in the decapod LCA sequence, the postnaupliar longitudinal muscle precursors [*lmp-mx1*], [*lmp-mx2*], [*lmp-t1*], and [*lmp-post*] occur simultaneously before offset of segment formation [FS] and formation of the postnaupliar appendage muscle precursors [*mx1-m*, *mx1-l*, *mx2-m*, *mx2-l*, *t1-m*, *t1-l*].

#### S. ingentis (Dendrobranchiata)

**Figure 12:**
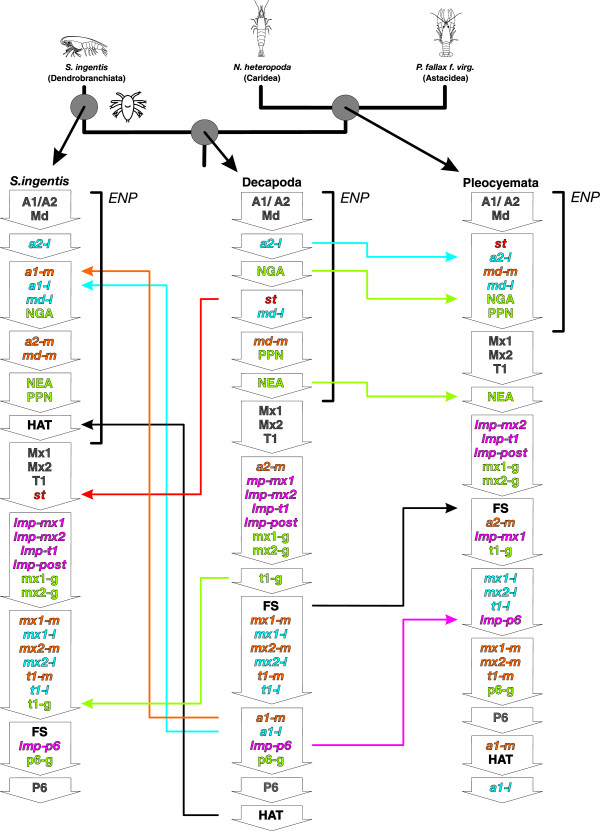
**Heterochronic changes and ancestral developmental sequences within Decapoda.** Phylogeny of Malacostraca as in Figure 11, but showing only Decapoda. Ancestral ontogenetic sequences reconstructed by heterochrony analysis using PGi are shown for the decapod and the pleocyemate LCA, as well as the terminal sequence of *S. ingentis* (Dendrobranchiata). Abbreviations: ***ENP*** *egg nauplius phase*. Event abbreviations are listed in Table 1.

Reconstructed ancestral event sequences of Decapoda, Pleocyemata, and the terminal developmental sequence of *S. ingentis* are given together with the heterochronic changes in Figure 12. In the lineage leading to *S. ingentis*, formation of the first antennal muscle precursors [*a1-m*, *a1-l*] and the hatching event [HAT] are shifted early. Formation of the stomodeal muscle precursor [*st*] is shifted to a later position after the *egg nauplius phase.* The simultaneous formation of postnaupliar appendage buds [Mx1], [Mx2], [T1], followed by postnaupliar longitudinal muscles and postnaupliar extrinsic appendage muscles is retained in the lineage leading to *S. ingentis*. Only formation of the first thoracic ganglion anlage [t1-g] is delayed.

#### Pleocyemata

In the pleocyemate stem lineage formation of the second antennal muscle precursor [*a2-l*], the naupliar ganglion anlagen [NGA] and the nauplius eye anlage [NEA] are shifted to a later position. The nauplius eye anlage [NEA] is therefore formed only after the *egg nauplius phase* as a result*.* Offset of segment formation [FS] and formation of the longitudinal muscle precursor in the sixth pleon segment [*lmp-p6*] are shifted to an earlier position.

#### Peracarida

**Figure 13:**
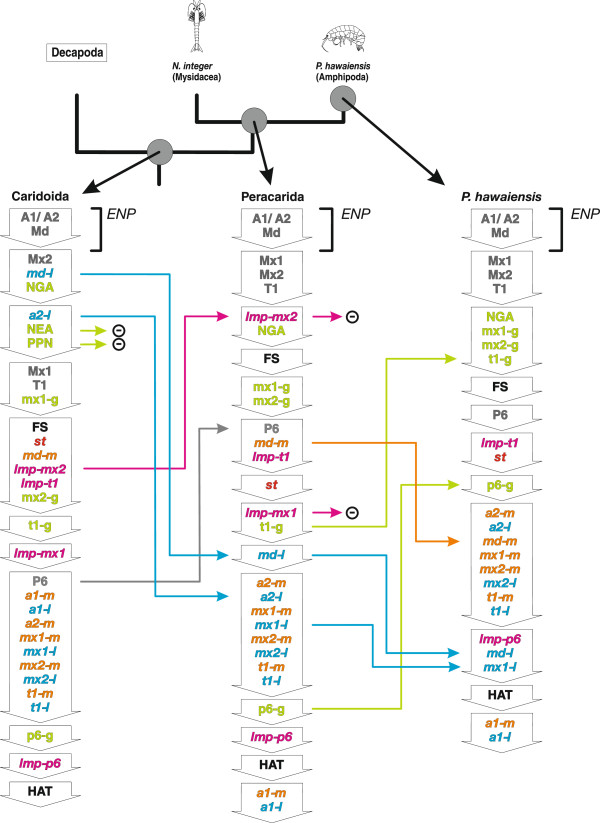
**Heterochronic changes and ancestral developmental sequences within Peracarida.** Phylogeny of malacostraca as in Figures 11 and 12. Only the caridoid LCA, the peracarid LCA, and the terminal sequence of *P. hawaiensis* are shown. Abbreviations: ***ENP*** *egg nauplius phase*. Event abbreviations are listed in Table 1.

The reconstructed ancestral sequences of the caridoid and peracaridan LCA, as well as the developmental sequence of *P. hawaiensis* are shown in Figure 13, and the respective changes of event positions are indicated. In the lineage leading to Peracarida, the nauplius eye anlage [NEA] and the posterior pioneer neurons [PPN] are lost. Formation of extrinsic appendage muscle precursors of the second antenna [*a2-l*] and the mandible [*md-l*] are shifted to a later position while the formation of the longitudinal muscle precursor [*lmp-mx2*] and the sixth pleopod bud [P6] are shifted to an earlier position. In the peracarid LCA sequence, the *egg nauplius phase* now only consists of epidermal appendage bud formation. It is followed by rapid formation of the postnaupliar appendage buds. The first part of the developmental sequence is dominated by formation of appendage buds and ganglion anlagen which all occur in strict anteroposterior progression. The majority of myogenic events is concentrated in the second half of the sequence and shows no trace of an anteroposterior gradient in development.

#### P. hawaiensis (Amphipoda)

*P. hawaiensis* shows loss of the longitudinal muscle precursors [*lmp-mx1*] and [*lmp-mx2*] in the first and second maxilla segments. Formation of extrinsic appendage muscle precursors [*md-m*], [*md-l*], and [*mx1-l*] is shifted late while formation of the first thoracic and sixth pleon ganglion anlagen [t1-g] and [p6-g] are shifted to earlier positions. As a result, appendage bud and ganglion anlage formation are even more concentrated at the beginning of the sequence. A temporal gap between formation of naupliar and postnaupliar features is shown only for appendage buds, but not for neurogenic or myogenic events.

## Discussion

### Tissue-related evolution of developmental timing

Studies on developmental genetics of the fruit fly *Drosophila melanogaster*[98] and experimental developmental studies on *P. hawaiensis*[99] suggest that the ectoderm has a strong regulatory influence on the development of the mesoderm in Arthropoda, but not vice versa. This does not necessarily imply that heterochronies within the ectoderm are unlikely. Fritsch & Richter [12] describe several instances of intraectodermal heterochrony in evolution of Branchiopoda. Yet tracing the evolutionary history of eumalacostracan, developmental timing by PGi in our study showed that the different tissue types (epidermis, nervous system, musculature) have taken different evolutionary paths depending on the germ layer they originate from. The mean heterochrony rates of ectoderm and mesoderm development that were calculated from the results of PGi analysis differ strongly (Table 4). Ectodermal development is generally less affected by heterochrony in malacostracan evolution than mesodermal development. Almost no heterochronic events appear in the naupliar region if only the ectodermal development is considered. Heterochrony of muscle precursor formation is far more extensive in the first antennal, second antennal, and mandibular segments than neural development and formation of appendage buds (Figure 10b). This suggests that the divergent evolution of developmental timing between mesodermal and ectodermal tissues reflects a modular property of the crustacean developmental system as would be expected due to findings from genetic and experimental developmental biology [98, 99].

Reconstruction of the ancestral developmental sequence by computational heterochrony analysis with PGi suggests that the naupliar pattern, known as ‘*egg nauplius stage*’, was present in the last common ancestor of Eumalacostraca as combination of epidermal and neurogenic, but not muscle developmental patterns. In the last common ancestor of Caridoida and Peracarida, the reconstructed sequence shows only an ‘*epidermal egg nauplius’*. Formation of naupliar ganglion anlagen, however, occurs only slightly later in the sequence. The persistence of the very early timing of epidermal and neural naupliar events in these lineages could be explained as the result of a developmental constraint that limited the plasticity of developmental timing in ectodermal development compared to mesodermal development. This constraint would represent a modular property of the developmental regulatory system patterning the naupliar ectoderm, similar to the observations on other arthropods [98, 99]. Evolutionary alterations of timing in ectodermal development of the naupliar segments would thus have had a stronger impact on the developmental outcome and viability of the organism and therefore would have been more likely eliminated by selection, than timing alterations in mesoderm development. Of course this explanation is based only on cross-species comparison of timing patterns and not on experimental investigations of the developmental systems. Nevertheless, our findings support the hypothesis that heterochronic change of muscle development played a major role in evolutionary loss and reacquisition of the *nauplius* larva.

### Evolution of naupliar developmental patterns in Malacostraca

In arthropod development, commonly the material of a variable number of anterior segments is laid down in a different manner than following segments that are added posteriorly during development. This is reflected by the process of short germ development in embryogenesis or anamorphic postembryonic development [100], and references therein and represents a condition of the arthropod ground pattern. A naupliar developmental pattern, meaning that the material of the first antennal, second antennal, and mandibular segments is formed (more or less) simultaneously before the posteriorly following segments, can be understood as a specialized form of the arthropod developmental pattern. The plesiomorphic condition for crustaceans (or Tetraconata) including extinct representatives of the stem lineage (called Crustacea *sensu lato* in [101]) was a ‘head’ larva with functional first antennae followed by three pairs of appendages. A larva bearing three appendage pairs—the *nauplius*—is considered apomorphic for crown-group Tetraconata [102] (Pancrustacea) (Eucrustacea or Crustacea *sensu stricto* in [101]) [17]. Our analysis reveals an ancestral developmental sequence for the branchiopod/malacostracan clade with an extensive *egg nauplius phase*. However, the reconstructed sequence is not fully compatible with a developmental mode comprising a free-swimming *nauplius* larva because some naupliar myogenic events are formed only after the *egg nauplius phase* and hatching occurs only after of the second maxilla and first thoracopod bud are formed. Yet a large number of postnaupliar developmental events, such as formation of postnaupliar ganglion anlagen and muscle precursors, occur after hatching which is in line with *nauplius* larva formation. Since a *nauplius* larva is predominant throughout the remaining crustacean taxa, we suggest that this was also the condition in the last common branchiopod/malacostracan ancestor and that the late position of many naupliar events is an artifact caused at this basal node by the extensive variation in the data set.

The developmental sequence of the malacostracan ground pattern could not be reconstructed, because Leptostraca, the sister group of Eumalacostraca according to the phylogeny of Richter & Scholtz [28], is not present in our taxon sampling. Embryogenesis of the leptostracan *Nebalia bipes* has been described [52, 53, 103] but unfortunately not sufficiently to integrate this species into the analysis. It is known however that *N. bipes* lacks free-swimming larval phases. Also, an *egg nauplius* stage in appendage morphogenesis is described for this species. The developmental pattern of the malacostracan last common ancestor can therefore not be expected to differ much from the eumalacostracan last common ancestor in these respects.

The eumalacostracan last common ancestor, according to heterochrony analysis with PGi, possessed a developmental sequence with late position of the hatching event and thus major postnaupliar developmental events occurring in embryogenesis. The late shift of the hatching event to the end of the sequence, the early shift of postnaupliar muscle precursor, ganglion anlagen, and appendage bud formation suggests that in the lineage leading to Malacostraca a change of ontogenetic mode took place and the *nauplius* larva was lost. Our analysis suggests that only formation of naupliar appendage buds and naupliar ganglia remained part of the *egg nauplius phase*. The *egg nauplius stage*[67, 69] was thus likely restricted to ectodermal tissues already in the eumalacostracan ground pattern.

Along the branches leading from the eumalacostracan to the caridoid and to the decapod last common ancestor, comparatively few heterochronies are recovered by our analysis. Formation of two naupliar muscle precursors is shifted to an earlier position while naupliar appendage bud formation and formation of naupliar ganglia are retained close to the beginning of the sequence. The developmental sequence reconstructed for the ground pattern of Decapoda shows an extensive *egg nauplius phase*, comprising all naupliar ectodermal events and formation of part of the naupliar muscle precursor group. Thus naupliar myogenic events must have been added to the *egg nauplius stage* in development before the emergence of a *nauplius* larva in the evolution of Decapoda.

The lineage leading to Dendrobranchiata represents a change in developmental mode and evolution of a free-swimming *nauplius* larva. Compared to the eumalacostracan stem lineage where the *nauplius* larva was lost, *S. ingentis* shows only few (five) heterochronies, of which only three are relevant for the reacquisition of the *nauplius* larva: early shift of the two muscle precursors of the first antenna and early shift of the hatching event. These changes were sufficient for the reacquisition of a free-swimming *nauplius* larva because the other necessary events were already in place in the developmental sequence of the decapod ground pattern. This refers to the naupliar appendage bud formation and formation of naupliar ganglia, which constitute the ectodermal *egg nauplius stage* in embryogenesis, as well as the early positions of naupliar myogenic events that are the result of heterochronic shifts in the lineages leading to Decapoda. Therefore, our results support the hypothesis formulated by Scholtz [67] that an embryonic *egg nauplius* served as a prerequisite for the secondary evolution of the dendrobranchiate *nauplius* larva.

Within Decapoda, in the lineage leading to Pleocyemata, an *egg nauplius* pattern in ectodermal development has been retained, together with a set of naupliar muscle precursors in the *egg nauplius* phase, according to our results. This pattern differs only minimally from the decapod ground pattern. Yet timing of naupliar myogenesis is altered within the Pleocyemata in the lineages leading to *N. heteropoda* and *P. fallax forma virginalis.* We should note that both species are direct developers, which is a derived condition within decapods. Yet the results of PGi suggest that the pleocyemate ground pattern is reminiscent of the decapod ground pattern and that alterations to myogenesis have occurred only within the group. Adding taxa with a more basal phylogenetic position and a *zoea-like* larva in future studies can be expected to uncover a similar condition.

It should be noted that the other malacostracan taxon with a *nauplius* larva, the Euphausiacea, is not represented in our study. Following the phylogeny used here, Euphausiacea are the sister group of Neocarida. Another popular phylogenetic hypothesis places Euphausiacea and Decapoda together in a monophylum called Eucarida [104, 105]. Thus mapping timing data on these two alternative hypotheses could improve our understanding of malacostracan phylogeny and clarify whether a *nauplius* larva evolved once or twice within Malacostraca. It is unlikely however that inclusion of Euphausiacea will significantly change the reconstructed developmental mode in the eumalacostracan or malacostracan ground pattern, as this depends on the position of other not included taxa, such as Leptostraca, Amphionidacea, and Syncarida.

### Evolution of a *zoea-like*larva

In Malacostraca, both ectodermal and mesodermal tissues, from the second maxillary to the sixth pleonal segment, are formed sequentially by proliferation of stem-like cells (ectoteloblasts and mesoteloblasts) in the posterior growth zone [61, 62, 106, 107]. This mechanism leads to an observable anteroposterior gradient of segment differentiation in the germ band. We have recently described a growth zone independent muscle precursor ‘*lmp-post*’ which likely plays a crucial role in development of a *zoea-like* larva [67, 73]. *lmp-post* is formed in the telson and extends anteriorly while at the same time longitudinal muscle precursors form in the anterior postnaupliar segments. This way a continuous longitudinal muscle strand across all trunk segments is formed, even before the full set of trunk segments is differentiated. *Zoea-like* larval forms share a functional, movable trunk, consisting of the thoracic or pleonal segments and a paddle-shaped telson which can perform extension and flexion movements, and actively participate in swimming, e.g., by performing tail flip escape reactions. Activity of [*lmp-post*] allows trunk functionality before the posterior trunk segments are fully developed, as is the case in *zoea-like* larvae. Further common features of *zoea-like* larva development in terms of developmental timing are rapid formation of appendages in an anterior set of postnaupliar segments (first and second maxillae, thoracopods), late formation of pleonal segments [P6], [P6-g], [*lmp-p6*] (with the exception of the stomatopod *pseudozoea*), and late offset of segment formation in the germ band [FS]. Event data for the second thoracic to the fifth pleonal segment was not analyzed because the data could not be acquired for a sufficient amount of species and semaphoronts.

The scenario reconstructed by PGi suggests that a *zoea-like* larva likely evolved independently in the lineages leading to Stomatopoda and Decapoda and that the eumalacostracan last common ancestor developed directly. The posterior longitudinal muscle primordium [*lmp-post*] which we consider a necessary feature for *zoea-like* larval motility was acquired twice independently in the lineages leading to Stomatopoda and Decapoda and was not part of the eumalacostracan ground pattern. The appendage bud and longitudinal muscle precursor of the sixth pleonal segment are formed early in the sequence reconstructed for the eumalacostracan last common ancestor, suggesting that functionality of the trunk did not precede differentiation in the posterior pleon segments, and consequently that direct development rather than a *zoea-like* larva constituted the developmental mode. We point out that the conclusions on this early node should be treated with caution because variation in the data set is extensive and also because we cannot rule out the possibility of bias due to the strong representation of direct development in the analysis.

For the last common ancestor of Decapoda, a *zoea-like* larva as hatching stages appears well supported. In the developmental sequence of the decapod ground pattern, appendage bud formation and formation of the longitudinal muscle precursor in the sixth pleonal segment occur close to the end of the sequence. Offset of segment formation also occurs late while the anterior postnaupliar appendage buds are formed simultaneously and just after the *egg nauplius phase.* Also all anterior longitudinal muscle precursors and *lmp-post* are formed simultaneously. The same is true for the extrinsic appendage muscle precursors of the anterior postnaupliar segments. In the lineage leading to Dendrobranchiata, timing of postnaupliar events relevant to *zoea-like* larva formation remains nearly unchanged (with the exception of [t1-g]). The evolution of the novel developmental mode of Dendrobranchiata, involving the novel larval stages *metanauplius*, *protozoea* and *mysis* stages, from an ancestral condition with a more extensive embryonic period did not depend on changes in developmental timing of the analyzed morphogenetic events. It is the predisplacement of the hatching event that makes the actual difference between larval and embryonic development, while the sequence in which appendage buds, ganglion anlagen, and muscle precursor are generated remains largely unchanged. Certainly, acceleration of differentiation processes which follow the formation of appendage, ganglion, or muscle anlagen in the segments of a viable free-swimming larva must be assumed for the evolution of the dendrobranchiate ontogenetic mode. In the lineage leading to Pleocyemata, [FS] and [*lmp-p6*] are shifted to earlier positions. Both changes point toward loss of *zoea-like* larva formation, but the majority of relevant events is still in place. Both pleocyemate representatives (*N. heteropoda* and *P. fallax forma virginalis*) develop directly and lack larval stages. Therefore, we consider early placement of [FS] and [*lmp-p6*] as bias toward direct development in our data set, not necessarily as part of the pleocyemate ground pattern.

### Evolution of developmental timing in Peracarida

Peracaridan development is derived in many respects relative to the malacostracan ground pattern, because of the advanced mode of brood care that is autapomorphic to this group [20, 28, 108–110]. In Peracarida, females possess a ventral brood pouch (marsupium), in which eggs are reared. *Nauplius* or *zoea-like* larvae are not found in peracarids. Changes to the developmental sequence in the peracarid stem lineage comprise loss of the nauplius eye and the posterior pioneer neurons, as well as late shift of several naupliar and early shift of postnaupliar events. In the ancestral peracaridan developmental sequence, naupliar and anterior postnaupliar appendage buds are formed rapidly at the beginning of the sequence, while naupliar ganglia are formed late. Offset of segment formation and formation of the posteriormost pleonal appendage bud occur early while the majority of muscle precursors are formed late. These properties of the developmental sequence do not resemble *zoea-like* developmental timing patterns. The condition in the caridoid ground pattern is difficult to interpret in terms of developmental mode. The late position of pleonal events suggests *zoea-like* larva formation but the posterior longitudinal muscle primordium [*lmp-post*] is not formed in the sequence. The question whether direct development might be plesiomorphic for Peracarida can therefore not be answered at this point.

Within Peracarida, most likely development was consistently adapted to efficient formation of juvenile body morphology after the advent of the new developmental mode, which was constrained by the specialized mode of maternal brood care. In *P. hawaiensis* (Amphipoda) the adaptation to efficient formation of juvenile body structure is intensified. Here the events of the six anterior segments appear in an order corresponding to the tissues they belong to: appendage buds, followed by ganglion anlagen, followed by muscle precursors. Together with the early offset of segment formation, this suggests a strong acceleration of morphogenesis in this lineage, which resulted in more rapid anteroposterior progression of segment formation and an earlier onset of tissue differentiation.

In Mysidacea, an inert larval stage hatches, and remains in the marsupium, a situation we call pesudodirect development. The hatchling is termed ‘*nauplioid*’ [110, 111]. The name is suggestive of a cryptic larval stage related to a *nauplius* larva. Also the early hatching event, presence of a solid cuticle with setation, and intramarsupial molting to the ‘postnauplioid’ stage suggest that a part of an ancestral larval developmental program is still active in Mysidacea. The evolutionary scenario reconstructed with PGi suggests that the mysid sequence is derived from an ancestor with only an epidermal *egg nauplius* phase and a late position of the hatching event. This may be an artifact of insufficient taxon sampling. However, Mysidacea show a unique timing pattern of appendage bud development with the first and second antennal bud being formed clearly before the mandible bud, early offset of segment formation, and finally late formation of naupliar ganglia and musculature. These observations suggest that the developmental pattern found in Mysidacea is not homologous to the *egg nauplius* pattern of Eumalacostraca.

## Conclusions

Our reconstruction of developmental sequence evolution of Malacostraca revealed that development of musculature has played a crucial role in evolutionary transitions between larval and embryonic development. The following conclusions can be drawn from our analysis of heterochrony:

- The eumalacostracan last common ancestor has retained the developmental timing pattern of *nauplius* larva formation in epidermal appendage development and neurogenesis, but not in myogenesis. The ontogenetic mode using a *nauplius* larva was replaced most likely by direct development in the lineage leading to the Malacostraca by delay in naupliar muscle development.
- Secondary evolution of the dendrobranchiate *nauplius* larva involved only little heterochronic change, because the major features of *naupliar* development were present already in the decapod last common ancestor. The transition relied on early shift of naupliar muscle precursors.
- According to our analysis, convergent evolution of a *zoea-like* larva in the stomatopod and decapod lineage is more likely than a *zoea-like* larva in the eumalacostracan last common ancestor.
- The developmental sequence of the peracarid last common ancestor has lost the larva-related timing patterns in embryogenesis. Developmental timing was likely adapted to efficient formation of juvenile body structure under the constraint of specialized brood care within the Peracarida.

Some key taxa of Malacostraca have not been sampled here: Leptostraca, Anaspidacea, Bathynellacea, and Euphausiacea. Also inclusion of additional event data, considering the thoracic and pleonal segments, more advanced stages of tissue differentiation, or the formation of external cuticular structures would contribute to a more detailed picture of malacostracan developmental evolution. Such investigations have the potential to further clarify the evolutionary history of malacostracan development, but this is left to future studies.

## Electronic supplementary material

Additional file 1: **Event sequence overview for all investigated taxa.** For each of the investigated taxa, a matrix is given in form of a spreadsheet, in which the events are listed against the specified semaphoronts. Specifications of semaphoronts used in our data set are given in bold in the upper row. Stage specifications from the literature are given in italics (second row). Rank values are also shown. Event categories, event abbreviations (brackets), and event numbers are given in three columns on the left. Furthermore, the literature source used to code the respective events is given. If an event occurs for the first time in a specific semaphoront, the respective field is marked with one of the following abbreviations or symbols: ‘*figure number*’ (events that are coded from observations made in the present study and depicted in the specified figure), ‘x’ (events that are coded from our observations but not depicted), ‘-’ (the event is not observed), ‘n’ (the event is not applicable), ‘[*reference number*]’ (the event is coded from the given literature source), ‘*scientists name*, personal communication’ (if the event is coded following personal communications). (XLSX 29 KB)

Additional file 2: **Nexus file for analysis of heterochrony.** Nexus file containing the rank-matrix and the tree topology. The data is formatted for use with PGi under R’s *ape* package. A rank value is coded for every event shown in Table 1. This yields a matrix of 7 × 33 cells. Since PGi cannot handle values with multiple digits, ranks 10, 11, and 12 were coded as letters a, b, and c, respectively. Absent data is coded as ‘z’ by convention. The tree topology from [28] is included in the nexus file, in a simplified form, which includes only the seven investigated taxa. (TXT 2 KB)

Additional file 3: **PGi superconsensus tree.** Superconsensus tree generated by PGi-analysis from pseudoconsensus trees of three independent runs with algorithm parameters set to 100 cycles of selection per node, 200 sequences per cycle of selection, a maximum of 100 ancestral developmental sequences to be retained at each node and ‘semi-exhaustive’ pseudoconsensus setting with a limit of 3,000 evaluated solutions of equal score. Developmental sequences in the superconsensus tree are shown using only the event numbers (shown in Table 1). Simultaneous events are combined by brackets; subsequent events are separated by a comma between brackets. The reconstructed ancestral developmental sequences are shown as plain text in boxes for every ancestral node. Terminal sequences are given in italics. Heterochronic events are shown by event numbers for every branch. A marks accelerated events; D marks delayed events. Bootstrap support values for every single heterochrony are given in parentheses. (PDF 1 MB)

## Abbreviations

A1: Anlage of the first antenna
*a1-l*: Lateral extrinsic muscle precursor of second antenna
*a1-m*: Medial extrinsic muscle precursor of first antenna
A2: Anlage of the second antenna
*a2-l*: Lateral extrinsic muscle precursor of second antenna
*a2-m*: Medial extrinsic muscle precursor of second antenna
FS: (Event) ‘Full segments,’ all mesodermal segment anlagen are present, offset of segment formation)
HAT: Hatching event, larva or Juvenile hatches from the egg membrane
LCA: Last common ancestor
*lmp-mx1*: Longitudinal muscle precursor in first maxilla segment
*lmp-mx2*: Longitudinal muscle precursor in second maxilla segment
*lmp-t1*: Longitudinal muscle precursor in first thoracopod segment
*lmp-p6*: Longitudinal muscle precursor in sixth pleopod segment
*lmp-post*: Posterior longitudinal muscle primordium
Md: Anlage of the mandible
*md-l*: Lateral extrinsic muscle precursor of mandible
*md-m*: Medial extrinsic muscle precursor of mandible
Mx1: Anlage of the first maxilla
*mx1-l*: Lateral extrinsic muscle precursor of first maxilla
*mx1-m*: Medial extrinsic muscle precursor of first maxilla
Mx2: Anlage of second maxilla
*mx2-l*: Lateral extrinsic muscle precursor of second maxilla
*mx2-m*: Medial extrinsic muscle precursor of second maxilla
NEA: Anlage of the nauplius eye
NGA: Anlagen of naupliar ganglia
T1: Anlage of the first thoracopod
t1-g: Anlagen of first thoracopod ganglion
*t1-l*: Lateral extrinsic muscle precursor of first thoracopod
*t1-m*: Medial extrinsic muscle precursor of first thoracopod
P6: Anlage of the sixth pleopod
p6-g: Anlagen of sixth pleopod ganglion
PGi: *Parsimov-based genetic inference*
PPN: Posterior pioneer neurons
*St*: Stomodeal muscle precursor group.

## References

[1] Gould SJ (1977). Ontogeny and phylogeny.

[2] McNamara KJ, McKinney ML (2005). Heterochrony, disparity, and macroevolution. Paleobiology.

[3] Alberch P (1980). Ontogenesis and morphological diversification. Integr Comp Biol.

[4] Harrington SM, Harrison LB, Sheil CA (2013). Ossification sequence heterochrony among amphibians. Evol Dev.

[5] Germain D, Laurin M (2009). Evolution of ossification sequences in salamanders and urodele origins assessed through event-pairing and new methods. Evol Dev.

[6] Weisbecker V, Mitgutsch C (2010). A large‒scale analysis of heterochrony in anuran cranial ossification patterns. J Zool Syst Evol Res.

[7] Velhagen WAJ (1997). Analyzing developmental sequences using sequence units. Syst Biol.

[8] Maxwell EE, Harrison LB, Larsson HCE (2010). Assessing the phylogenetic utility of sequence heterochrony: evolution of avian ossification sequences as a case study. Zoology.

[9] Sánchez-Villagra MR, Goswami A, Weisbecker V, Mock O, Kuratani S (2008). Conserved relative timing of cranial ossification patterns in early mammalian evolution. Evol Dev.

[10] Jeffery JE, Bininda-Emonds ORP, Coates MI, Richardson MK (2002). Analyzing evolutionary patterns in amniote embryonic development. Evol Dev.

[11] Bininda-Emonds ORP, Jeffery JE, Sánchez-Villagra MR, Hanken J, Colbert M, Pieau C (2007). Forelimb-hindlimb developmental timing changes across tetrapod phylogeny. BMC Evol Biol.

[12] Fritsch M, Bininda-Emonds OR, Richter S (2013). Unraveling the origin of Cladocera by identifying heterochrony in the developmental sequences of Branchiopoda. Front Zool.

[13] Smirthwaite JJ, Rundle SD, Bininda-Emonds ORP, Spicer JI (2007). An integrative approach identifies developmental sequence heterochronies in freshwater basommatophoran snails. Evol Dev.

[14] Haug JT, Maas A, Waloszek D (2010). †*Henningsmoenicaris scutula*, †*Sandtorpia vestrogothiensis* gen. et sp. nov. and heterochronic events in early crustacean evolution. Earth Env Sci T R So.

[15] Sumrall CD, Wray GA (2007). Ontogeny in the fossil record: diversification of body plans and the evolution of “aberrant” symmetry in Paleozoic echinoderms. Paleobiology.

[16] Webster M, Zelditch ML (2005). Evolutionary modifications of ontogeny: heterochrony and beyond. Paleobiology.

[17] Walossek D, Müller KJ (1990). Upper Cambrian stem-lineage crustaceans and their bearing upon the monophyletic origin of Crustacea and the position of *Agnostus*. Lethaia.

[18] Lauterbach KE (1986). Zum Grundplan der Crustacea. Verh Natwiss Ver Hambg.

[19] Anderson D, Kerkut GA (1973). Crustaceans. Embryology and phylogeny of annelids and arthropods.

[20] Gruner HE (1993). Unterklasse Malacostraca. Lehrbuch der Speziellen Zoologie Band I, 4 Tl.

[21] Martin JW, Criales MM, Dos Santos A, Martin J, Olesen J, Høeg JT (2014). Dendrobranchiata. Atlas of Crustacean Larvae.

[22] Martin JW, Gomez-Gutierrez J, Martin J, Olesen J, Høeg JT (2014). Euphausiacea. Atlas of Crustacean Larvae.

[23] Siewing R (1956). Untersuchungen zur Morphologie der Malacostraca. Zool Jb Anat.

[24] Richter S (1999). The structure of the ommatidia of the Malacostraca (Crustacea) - a phylogenetic approach. Verh Natwiss Ver Hambg.

[25] Schram FR, Hof CHJ, Edgecombe GD (1998). Fossils and the interrelationships of major crustacean groups. Arthropod Fossils and Phylogeny.

[26] Wills M (1998). Crustacean disparity through the Phanerozoic: comparing morphological and stratigraphic data. Biol J Linn Soc.

[27] Ax P (1999). Das System der Metazoa II.

[28] Richter S, Scholtz G (2001). Phylogenetic analysis of the Malacostraca (Crustacea). J Zool Syst Evol Res.

[29] Spears T, Abele LG, Fortey RA, Thomas RH (1997). Crustacean phylogeny inferred from 18S rDNA. Arthropod Relationships.

[30] Spears T, Abele LG (1999). Phylogenetic relationships of crustaceans with foliaceous limbs: an 18 s rDNA study of Branchiopoda, Cephalocarida, and Phyllocarida. J Crust Biol.

[31] Jenner RA, Ní Dhubhghaill C, Ferla MP, Wills MA (2009). Eumalacostracan phylogeny and total evidence: limitations of the usual suspects. BMC Evol Biol.

[32] Von Reumont BM, Jenner RA, Wills MA, Dell’ampio E, Pass G, Ebersberger I (2012). Pancrustacean phylogeny in the light of new phylogenomic data: support for Remipedia as the possible sister group of Hexapoda. Mol Biol Evol.

[33] Ahyong ST, Haug JT, Haug C, Martin J, Olesen J, Høeg JT (2014). Stomatopoda. Atlas of Crustacean Larvae.

[34] Manning RB, Provenzano AJJ (1963). Studies on development of stomatopod Crustacea 1. Early larval stages of *Gonodactylus oerstedii* HANSEN. Bull Mar Sci Gulf Caribean.

[35] Provenzano J, Manning RB (1978). Studies on development of stomatopod Crustacea 2. The later larval stages of *Gonodactylus oerstedii* HANSEN reared in the laboratory. Bull Mar Sci.

[36] Shanbhogue SL (1978). The embryonic and early larval development of *Gonodactylus falcatus* (FORSSKAL) (Crustacea: Stomatopoda) from India. J Mar Biol Assoc India.

[37] Dobkin S (1961). Early developmental stages of the pink shrimp *Penaeus duorarum*. Fish Bull United States Fish Wildl Serv.

[38] Heegard P, Heegard P (1969). Larvae of decapod Crustacea - the Amphionidae. The Carlsberg Foundation’s Oceanographical Expedition Round the World 1928–1930.

[39] Dobkin S (1963). The larval development of *Palaemonetes paludosus* (GIBBES, 1950) (Decapoda, Palaemonidae), reared in the laboratory. Crustaceana.

[40] Gurney R (1936). Larvae of decapod Crustacea. Discov Rep.

[41] Goy JW, Martin J, Olesen J, Høeg JT (2014). Stenopodidea. Atlas of Crustacean Larvae.

[42] Guerao G, Cuesta JA, Martin J, Olesen J, Høeg JT (2014). Caridea. Atlas of Crustacean Larvae.

[43] Martin JW, Kutschera V, Martin J, Olesen J, Høeg JT (2014). Amphionidacea. Atlas of Crustacean Larvae.

[44] Jakobi H (1954). Biologie und postembryonale Entwicklung von *Antrobathynella stammeri*. Zool Jb Syst.

[45] Jakobi H (1958). Biologie, Entwicklungsgeschichte und Systematik von *Bathynella natans* VEJD. Zool Jb Syst.

[46] Schminke HK, Martin J, Olesen J, Høeg JT (2014). Syncarida. Atlas of Crustacean Larvae.

[47] Knight M (1978). Larval development of *Euphausia fallax* HANSEN (Crustacea: Euphausiacea) with a comparison of larval morphology within the *E. gibboides* species group. Bull Mar Sci.

[48] Knight MD (1976). Larval development of *Euphausia sanzoi* TORELLI (Crustacea: Euphausiacea). Bull Mar Sci.

[49] Zimmer C, Gruner HE (1956). Euphausiacea. Klassen und Ordnungen des Tierreichs.

[50] Mauchline J, Fisher LR (1969). The biology of euphausiids. Mar Biol.

[51] Ahyong ST, Harling C (2000). The phylogeny of the stomatopod Crustacea. Aust J Zool.

[52] Manton SM (1934). On the embryology of the crustacean *Nebalia bipes*. Philos Trans R Soc Ser B.

[53] Olesen J, Walossek D (2000). Limb ontogeny and trunk segmentation in *Nebalia species* (Crustacea, Malacostraca, Leptostraca). Zoomorphology.

[54] Hickman V (1936). The embryology of the syncarid crustacean, *Anaspides tasmaniae*. Pap Proc R Soc Tasmania.

[55] Barker D (1962). A study of *Thermosbaena mirabilis* (Malacostraca, Peracarida) and its reproduction. Quart J Microsc Sci.

[56] Wolff C (2009). The embryonic development of the malacostracan crustacean *Porcellio scaber* (Isopoda, Oniscidea). Dev Genes Evol.

[57] Wolff C, Martin J, Olesen J, Høeg JT (2014). Amphipoda. Atlas of Crustacean Larvae.

[58] Boyko CB, Wolff C, Martin J, Olesen J, Høeg JT (2014). Isopoda and Tanaidacea. Atlas of Crustacean Larvae.

[59] Scholtz G (2000). Evolution of the nauplius stage in malacostracan crustaceans. J Zool Syst Evol Res.

[60] Shiino SM (1942). Studies on the embryology of *Squilla oratoria* DE HAAN. Mem Coll Sci Univ Kyoto Ser B.

[61] Öishi S (1960). Studies on the teloblasts in the decapod embryo II. Origin of teloblasts in *Pagurus samuelis* (STIMPSON) and *Hemigrapsus sanguineus* (DE HAAN). Embryologia.

[62] Öishi S (1959). Studies on the teloblasts in the decapod embryo I. Origin of teloblasts in *Heptacarpus rectirostris* (STIMPSON). Embryologica.

[63] Weygoldt P (1961). Beitrag zur Kenntnis der Ontogenie der Dekapoden: Embryologische Untersuchungen an *Palaemonetes varians* (LEACH). Zool Jb Anat.

[64] Scheidegger G (1976). Stadien der Embryonalentwicklung von *Eupagurus prideauxi* LEACH (Crustacea, Decapoda, Anomura) unter besonderer Berücksichtigung der Darmentwicklung und der am Dotterabbau beteiligten Zelltypen. Zool Jb Anat.

[65] Scholtz G (1992). Cell lineage studies in the crayfish *Cherax destructor* (Crustacea, Decapoda): germ band formation, segmentation, and early neurogenesis. Roux’s Arch Dev Biol.

[66] Zilch R (1974). Embryonalentwicklung von *Thermosbaena mirabilis* MONOD (Crustacea, Malacostraca, Pancarida). Zool Jb Anat.

[67] Jirikowski GJ, Richter S, Wolff C (2013). Myogenesis of Malacostraca - the “egg-nauplius” concept revisited. Front Zool.

[68] Alberch P, Gould S, Oster G, Wake D (1979). Size and shape in ontogeny and phylogeny. Paleobiology.

[69] Vilpoux K, Sandeman R, Harzsch S (2006). Early embryonic development of the central nervous system in the Australian crayfish and the Marbled crayfish (Marmorkrebs). Dev Genes Evol.

[70] Harrison LB, Larsson HC (2008). Estimating evolution of temporal sequence changes : a practical approach to inferring ancestral developmental sequences and sequence heterochrony. Syst Biol.

[71] Sears KE (2009). Differences in the timing of prechondrogenic limb development in mammals: the marsupial-placental dichotomy resolved. Evolution.

[72] Wirkner CS, Richter S (2010). Evolutionary morphology of the circulatory system in Peracarida. Cladistics.

[73] Jirikowski G, Kreissl S, Richter S, Wolff C (2010). Muscle development in the marbled crayfish - insights from an emerging model organism (Crustacea, Malacostraca, Decapoda). Dev Genes Evol.

[74] Wildt M, Harzsch S (2002). A new look at an old visual system: structure and development of the compound eyes and optic ganglia of the brine shrimp *Artemia salina* LINNEUS, 1758 (Branchiopoda, Anostraca). J Neurobiol.

[75] Frase T, Richter S (2013). The fate of the onychophoran antenna. Dev Genes Evol.

[76] Kiernan DA, Hertzler PL (2006). Muscle development in dendrobranchiate shrimp, with comparison with *Artemia*. Evol Dev.

[77] Hennig W (1966). Phylogenetic Systematics.

[78] Wolfe JM, Hegna TA (2013). Testing the phylogenetic position of Cambrian pancrustacean larval fossils by coding ontogenetic stages. Cladistics.

[79] Smith K (2001). Heterochrony revisited: the evolution of developmental sequences. Biol J Linn Soc.

[80] Alwes F, Scholtz G (2006). Stages and other aspects of the embryology of the parthenogenetic Marmorkrebs (Decapoda, Reptantia, Astacida). Dev Genes Evol.

[81] Browne WE, Price AL, Gerberding M, Patel NH (2005). Stages of embryonic development in the amphipod crustacean, *Parhyale hawaiensis*. Genesis.

[82] Fischer AHL, Scholtz G (2010). Axogenesis in the stomatopod crustacean *Gonodactylaceus falcatus* (Malacostraca). Invertebr Biol.

[83] Ungerer P, Geppert M, Wolff C (2011). Axogenesis in the central and peripheral nervous system of the amphipod crustacean *Orchestia cavimana*. Integr Zool.

[84] Benesch R (1969). Zur Ontogenie und Morphologie von *Artemia salina* (L.). Zool Jb Anat.

[85] Grobben K (1919). Über die Muskulatur des Vorderkopfes der Stomatopoden und die systematische Stellung dieser Malakostrakengruppe. Sitz Ber Akad Wiss Wien.

[86] Hertzler PL, Freas WR (2009). Pleonal muscle development in the shrimp *Penaeus* (*Litopenaeus*) *vannamei* (Crustacea: Malacostraca: Decapoda: Dendrobranchiata). Arthropod Struct Dev.

[87] Young JH (1959). Morphology of the white shrimp *Penaeus setiferus* ( LINNAEUS 1758). Fish Bull Fish Wild Serv.

[88] Schmidt W, Ehlers E (1915). Die Muskulatur von *Astacus fluviatilis* (*Potamobius astacus* L.). Zeitschrift für Wissenschaftliche Zoologie.

[89] Manton SM (1928). On the embryology of a mysid crustacean, *Hemimysis*. Philos Trans R Soc Lond B Biol Sci.

[90] Elofsson R (1965). The nauplius eye and frontal organs in Malacostraca (Crustacea). Sarsia.

[91] Weygoldt P (1958). Die Embryonalentwicklung des Amphipoden *Gammarus pulex pulex* (L). Zool Jb Anat.

[92] Maddison WP, Maddison DR (2011). Mesquite: A modular system for evolutionary analysis.

[93] Schulmeister S, Wheeler WC (2004). Comparative and phylogenetic analysis of developmental sequences. Evol Dev.

[94] Jeffery JE, Bininda-Emonds ORP, Coates MI, Richardson MK (2005). A new technique for identifying sequence heterochrony. Syst Biol.

[95] Paradis E, Claude J, Strimmer K (2004). APE : Analyses of phylogenetics and evolution in R language. Bioinformatics.

[96] R-Core-Team (2007). A language and environment for statistical computing.

[97] Richter S, Loesel R, Purschke G, Schmidt-Rhaesa A, Scholtz G, Stach T (2010). Invertebrate neurophylogeny: suggested terms and definitions for a neuroanatomical glossary. Front Zool.

[98] Rao Y, Vaessin H, Jan LY, Jan YN (1991). Neuroectoderm in *Drosophila* embryos is dependent on the mesoderm for positioning but not for formation. Genes Dev.

[99] Hannibal RL, Price AL, Patel NH (2012). The functional relationship between ectodermal and mesodermal segmentation in the crustacean, *Parhyale hawaiensis*. Dev Biol.

[100] Scholtz G, Wolff C, Minelli A, Boxhall G, Fusco G (2013). Arthropod Embryology: Cleavage and germ band development. Berlin.

[101] Waloszek D, Maas A (2005). The evolutionary history of crustacean segmentation: a fossil-based perspective. Evol Dev.

[102] Richter S (2002). The Tetraconata concept: hexapod-crustacean relationships and the phylogeny of Crustacea. Org Divers Evol.

[103] Pabst T, Scholtz G (2009). The development of phyllopodous limbs in Leptostraca and Branchiopoda. J Crust Biol.

[104] Wills MA, Fortey RA, Thomas RH (1998). A phylogeny of recent and fossil Crust derived from morphological characters. Arthropod Relationships.

[105] Watling L, Schram FR, von Vaupel Klein JC (1999). Towards understanding the relationship of the Peracaridan orders: the necessity of determining exact homologies. Crustaceans and the Biodiversity Crisis, Proc Fourth Int Crustacean Congress, Amsterdam.

[106] Dohle W, Scholtz G (1988). Clonal analysis of the crustacean segment: the discordance between genealogical and segmental borders. Development.

[107] Scholtz G, Dohle W (1996). Cell lineage and cell fate in crustaceans - a comparative approach. Int J Dev Biol.

[108] Calman WT (1909). Appendiculata, Crustacea. A treatise on zoology.

[109] Spears T, DeBry RW, Abele LG, Chodyla K (2005). Peracarid monophyly and interordinal phylogeny inferred from nuclear small-subunit ribosomal DNA sequences (Crustacea: Malacostraca: Peracarida). Proc Biol Soc Washingt.

[110] Wortham-Neal JL, Price WW (2002). Marsupial developmental stages in *Americamysis bahia* (Mysida: Mysidae). J Crust Biol.

[111] Wittmann KJ (1981). Comparative marsupial biology and morphology in *Leptomysis* and other mediterranean Mysidacea (Crustacea). J Exp Mar Biol Ecol.

