# Supplementary figures and images for: Evolution of eumalacostracan development—new insights into loss and reacquisition of larval stages revealed by heterochrony analysis
Source: EvoDevo. 2015 Mar 11;6:4. doi: 10.1186/2041-9139-6-4 (PMC4429915; doi:10.1186/2041-9139-6-4)

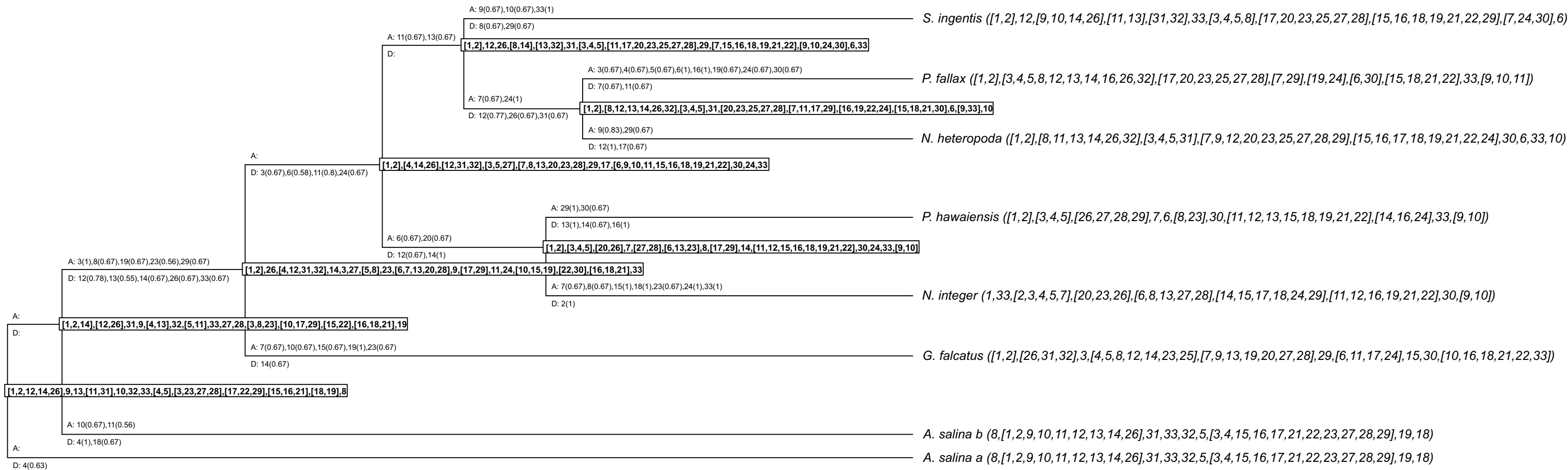

Supplement: Supplementary file 3 — Additional file 3: PGi superconsensus tree. Superconsensus tree generated by PGi-analysis from pseudoconsensus trees of three independent runs with algorithm parameters set to 100 cycles of selection per node, 200 sequences per cycle of selection, a maximum of 100 ancestral developmental sequences to be retained at each node and ‘semi-exhaustive’ pseudoconsensus setting with a limit of 3,000 evaluated solutions of equal score. Developmental sequences in the superconsensus tree are shown using only the event numbers (shown in Table 1). Simultaneous events are combined by brackets; subsequent events are separated by a comma between brackets. The reconstructed ancestral developmental sequences are shown as plain text in boxes for every ancestral node. Terminal sequences are given in italics. Heterochronic events are shown by event numbers for every branch. A marks accelerated events; D marks delayed events. Bootstrap support values for every single heterochrony are given in parentheses. (PDF 1 MB) [file 13227_2014_151_MOESM3_ESM.pdf]
